# The Use of Mobile Apps for Heart Failure Self-management: Systematic Review of Experimental and Qualitative Studies

**DOI:** 10.2196/33839

**Published:** 2022-03-31

**Authors:** Leticia Bezerra Giordan, Huong Ly Tong, John J Atherton, Rimante Ronto, Josephine Chau, David Kaye, Tim Shaw, Clara Chow, Liliana Laranjo

**Affiliations:** 1 Westmead Applied Research Centre Faculty of Medicine and Health University of Sydney Sydney Australia; 2 Department of Health Sciences Macquarie University Sydney Australia; 3 Department of Cardiology, Royal Brisbane and Women's Hospital and Faculty of Medicine University of Queensland Brisbane Australia; 4 Faculty of Medicine University of Queensland Brisbane Australia; 5 Alfred Hospital Baker Heart and Diabetes Institute Monash University Melbourne Australia; 6 Faculty of Medicine and Health University of Sydney Sydney Australia; 7 Australian Institute of Health Innovation Macquarie University Sydney Australia

**Keywords:** heart failure, self-management, mobile health, mobile app, secondary prevention, mobile phone

## Abstract

**Background:**

Heart failure self-management is essential to avoid decompensation and readmissions. Mobile apps seem promising in supporting heart failure self-management, and there has been a rapid growth in publications in this area. However, to date, systematic reviews have mostly focused on remote monitoring interventions using nonapp types of mobile technologies to transmit data to health care providers, rarely focusing on supporting patient self-management of heart failure.

**Objective:**

This study aims to systematically review the evidence on the effect of heart failure self-management apps on health outcomes, patient-reported outcomes, and patient experience.

**Methods:**

Four databases (PubMed, Embase, CINAHL, and PsycINFO) were searched for studies examining interventions that comprised a mobile app targeting heart failure self-management and reported any health-related outcomes or patient-reported outcomes or perspectives published from 2008 to December 2021. The studies were independently screened. The risk of bias was appraised using Cochrane tools. We performed a narrative synthesis of the results. The protocol was registered on PROSPERO (International Prospective Register of Systematic Reviews; CRD42020158041).

**Results:**

A total of 28 articles (randomized controlled trials [RCTs]: n=10, 36%), assessing 23 apps, and a total of 1397 participants were included. The most common app features were weight monitoring (19/23, 83%), symptom monitoring (18/23, 78%), and vital sign monitoring (15/23, 65%). Only 26% (6/23) of the apps provided all guideline-defined core components of heart failure self-management programs: education, symptom monitoring, medication support, and physical activity support. RCTs were small, involving altogether 717 participants, had ≤6 months of follow-up, and outcomes were predominantly self-reported. Approximately 20% (2/10) of RCTs reported a significant improvement in their primary outcomes: heart failure knowledge (*P*=.002) and self-care (*P*=.004). One of the RCTs found a significant reduction in readmissions (*P*=.02), and 20% (2/10) of RCTs reported higher unplanned clinic visits. Other experimental studies also found significant improvements in knowledge, self-care, and readmissions, among others. Less than half of the studies involved patients and clinicians in the design of apps. Engagement with the intervention was poorly reported, with only 11% (3/28) of studies quantifying app engagement metrics such as frequency of use over the study duration. The most desirable app features were automated self-monitoring and feedback, personalization, communication with clinicians, and data sharing and integration.

**Conclusions:**

Mobile apps may improve heart failure self-management; however, more robust evaluation studies are needed to analyze key end points for heart failure. On the basis of the results of this review, we provide a road map for future studies in this area.

## Introduction

### Background

Heart failure affects approximately 40 million people worldwide [1]. A diagnosis of heart failure portends a poor prognosis, with a 12-month mortality rate of 17% for patients who are hospitalized and 7% for patients who are stable or ambulatory [2]. Hospitalization is associated with a 3-fold increased risk of death [3,4] and is preventable with good quality self-management [4-6], including symptom monitoring and taking prompt action when deterioration begins [4,7]. However, there are several barriers to achieving good quality self-management, such as lack of knowledge, symptom recognition, motivation, and confidence [8]. Addressing these can improve outcomes; yet, delivering such models for support at scale is challenging.

Mobile health (mHealth)—medical and public health practice supported by mobile devices, such as mobile phones, patient monitoring devices, personal digital assistants, and other wireless devices [9]—has excellent potential for cardiovascular disease prevention [10-14]. In particular, app interventions seem promising as they can automate the self-monitoring of physiological data, facilitate symptom and medication tracking, and provide reminders and personalized feedback to promote patient engagement [15-17]. To date, no systematic reviews have focused exclusively on mobile apps to support self-management of heart failure. Previous mHealth systematic reviews on heart failure have mostly reported remote monitoring interventions using older technologies such as phone calls and interactive voice response to transmit data to health care providers, rarely focusing on supporting patient self-management [18-27]. A total of 3 nonsystematic reviews evaluated the content and quality of existing commercial heart failure apps and mHealth interventions without assessing their impact or patient perspectives [28-30].

### Aims

This systematic review aims to examine the role of mobile apps in heart failure self-management, specifically, their impact on improving (1) clinical outcomes, (2) patient-reported measures, and (3) self-management knowledge and behaviors and in addition, examine the acceptability and feasibility of these interventions, as well as patient perspectives, needs, and preferences for specific app features.

## Methods

### Database Search

A systematic search of the literature was performed in October 2019 and updated in December 2021 on PubMed, Embase, CINAHL, and PsycINFO, using several search terms such as mobile apps, heart failure, and self-management (Multimedia Appendix 1). The reference lists of relevant articles and gray literature such as dissertations, theses, and conference proceedings were also screened to ensure that all eligible studies were captured. The search was limited from 2008 onward as app stores were launched in that year [31]. No language limits were applied.

### Eligibility Criteria

Studies were included if they (1) focused on adult patients with heart failure, (2) involved an intervention comprising a mobile app to support heart failure self-management (ie, provision of education and support to increase patients’ skills and confidence in managing their disease [32])—the mobile app could be a single component in the intervention or be combined with other intervention components (eg, wireless devices for remote monitoring)—(3) included any type or no comparison (eg, qualitative studies), (4) reported impact on any health outcome or patient-reported measure (eg, self-management and medication adherence) or focused on patients’ perspectives, and (5) were a primary research study involving the use or testing of the mobile app intervention. Studies were excluded if they (1) did not involve the use of the app by patients with heart failure and (2) assessed interventions without a clear component of heart failure self-management (eg, patients using the app only to input data to be analyzed by health care professionals).

### Screening

The screening form was piloted by 2 investigators before beginning the screening process. The 2 investigators independently screened studies based on the information in their titles and abstracts and then performed the full-paper screening. Disagreements were resolved through discussion between the reviewers or by a third reviewer. Cohen κ statistic was used to measure intercoder agreement in the initial and full-text screening [33].

### Data Extraction and Synthesis

One of the reviewers extracted the following information from the included studies: author, year of publication, country, study design, sample size, population characteristics, study duration or intervention use time, intervention characteristics (eg, technology components and others, mobile app features, and presence or absence of personalization), comparison, outcomes, and main results. The 2 investigators reviewed the data extraction form for consistency. The coding of behavior change techniques (BCTs) according to the BCT taxonomy [34] was conducted by 1 researcher and reviewed by another. Studies’ quality and risk of bias were appraised by 2 researchers using Cochrane’s risk of bias tool [35] for randomized controlled trials (RCTs) and the *Risk Of Bias In Nonrandomized Studies of Interventions* [36] tool for other experimental studies. Disagreements were resolved by a third reviewer. We performed a narrative synthesis of the studies. The PRISMA (Preferred Reporting Item for Systematic Reviews and Meta-Analyses) 2020 statement was followed (Multimedia Appendix 2) [37], and the protocol was registered on PROSPERO (International Prospective Register of Systematic Reviews; CRD42020158041).

## Results

### Search and Screening Results

**Figure 1:**
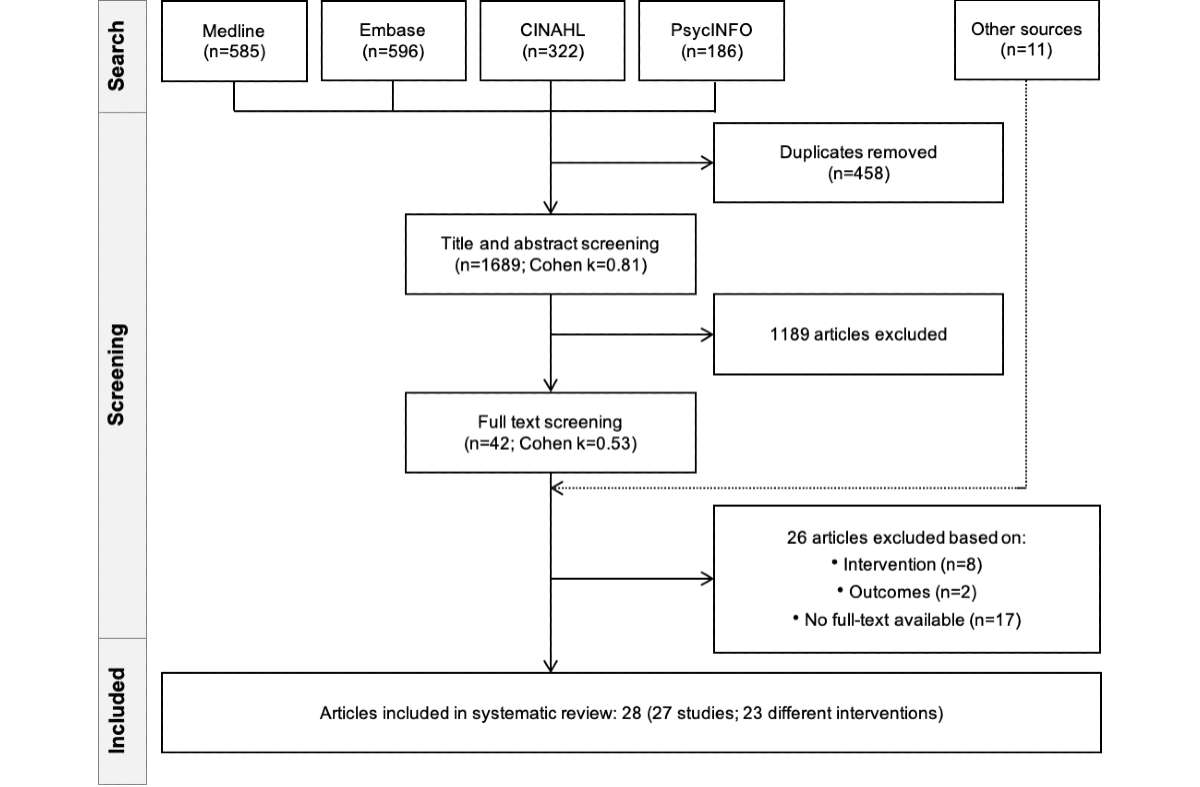
PRISMA (Preferred Reporting Items for Systematic Reviews and Meta-Analyses) ﬂow diagram of the study selection process.

The database search retrieved 1689 citations, from which 458 (27.1%) duplicates were removed (Figure 1). After title and abstract screening of the 1689 articles, 1189 (70.4%) were excluded. Full-text screening was conducted for 42 articles, and a further 26 (62%) papers were excluded (see Multimedia Appendix 3 for reasons for exclusion). A total of 12 additional papers were identiﬁed—1 (8%) from the reference list of the included studies and 11 (92%) from database alerts and search updates—leading to the inclusion of 28 articles [38-65] for ﬁnal analysis (corresponding to 27 studies, as 1 study was published in 2 different articles [38,65]). The Cohen κ statistic was 0.81 (excellent agreement) for the title and abstract screening and 0.53 (fair agreement) for the full-text screening before the consensus agreement was reached [66].

### Study Characteristics

All 28 included articles [38-65] were published from 2012 onward and covered 27 studies and 23 interventions (n=4, 17% interventions were evaluated in ≥1 paper, using different study designs [39,44,47,52,56,59,63-65]). Of the 28 studies, there were 18 (64%) experimental studies [38-55], (n=10, 56% RCTs [38-47] and n=8, 44% quasi-experimental [48-55]; n=7, 39% with a qualitative component [38,44,46,49,51,52,54]; Table 1), 9 (32%) qualitative-only studies, 5 (18%) that included interviews [56-60], and 4 (14%) that involved a survey with open-ended questions (Table 2) [57-64]. Most studies were conducted in the United States (15/28, 54%) [39,40,44,45,48,50,52,54,56,58,60-64] and Canada (4/28, 14%) [47,51,55,59], and most were single-center, except for a few (5/28, 18%) [38,42,43,55,62]. There were 1397 participants (n=8-232 in experimental studies and 5-37 in qualitative studies), mean age was 63.4 years, 30% were women, 68% were White (from 15/28, 54% studies that reported on ethnicity), and the average education level was high (Multimedia Appendix 4 [38-65]). The study duration in the experimental studies ranged from 2 weeks to 12 months (average of 3.2 months). The 10 RCTs had a moderate risk of bias [35]; the quasi-experimental studies were of lower quality (Multimedia Appendix 5 [36,38-55]) [35].

**Table 1:** Characteristics of experimental studies.

First author^a^		Study design	Follow-up (months)	Sample size (intervention; control)	Age (years), mean	Women (%)	Intervention	Control	Main results^b^
**RCTs^c^**									
	Clays et al [38,65]	RCT + interviews	6	65 (38; 23)	63	23	App + devices (weight, BP^d^, pill organizer, and wrist band): monitoring weight, BP, physical activity, and HR^e^; psychological support; education	Standard care	Between-groups: improvement in depression and anxiety measures (*P*<.001) NSf: between-groups quality of lifeg,h, self-careh,i, exercise capacity, illness perception Intervention group: increase in self-care (*P*<.05) and decrease in sexual problems (*P*<.05)
	Schmaderer et al [39]	RCT (3 arms)	3	74 (27; 26; 27)	56.3	54	App + wireless weight scale + Zoom visit with clinicians: monitoring medications and weight; automated feedback; graphical displays; education; clinician communication; reminders	App + wireless-weight scale: monitoring medications and weight	Between-groups: decrease in rehospitalization (*P*=.02) NS: quality of lifeh,j, EDk presentations, and hospitalizations
	Wei et al [40]	RCT + interviews	1.5	28 (15; 13)	63	25	App + wireless weight scale: monitoring weight; manual input of diet sodium, and exercise, symptoms; automated feedback; graphical displays; education; clinician communication	Standard care + written education materials	Intervention group: direct correlation between duration of app use and improvement in heart failure knowledgel (ρ=0.59; *P*=.04) and quality of life (ρ=0.63; *P*=.03)m Feasibilityh and engagement: in the intervention group, 5 patients logged ≥1 interaction with the app per day on average, and 2 patients logged an interaction with the app every other day on average.
	Yanicelli et al [41]	RCT	3	40 (20; 20)	52	20	Telemonitoring via app: monitoring (manual input) weight, BP, HR, and symptoms	Standard care	Between-groups: increase in self‑careh,n (*P*=.004) NS: medication adherenceh
	Rahimi et al [42]	RCT	6	202 (101; 101)	71.3	28	Telemonitoring via tablet app + devices (weight, BP, and HR): monitoring weight, BP, HR, and symptoms; automated feedback; EMR^o^ integration; graphical displays; education; clinician communication; reminders	Tablet app + devices; no clinician communication	Between-groups: decrease in systolic BP (*P*=.03) NS: achieving optimal medical therapyh and physical well-being (self-assessed NYHAp class)
	Wonggom et al [43]	RCT	3	36 (17; 19)	67.5	19	App with avatar: education	Standard care	Between-groups: increase in heart failure knowledgeh,q (*P*=.002) NS: self-caren; general practitioner visits, ED presentations, and hospital readmission
	Athilingam et al [44]	RCT + open-ended questionnaire	1	18 (9; 9)	53	56	App + chest-worn sensor: monitoring HR and physical activity, weight, and BP, and symptoms; automated feedback; graphical displays; medication adherence; education	Standard care	Between-groups: increase in self-care management (*P*=.01) and confidence (*P*=.03)n, heart failure knowledgel (*P*=.04); <50% used the app daily NS: quality of lifem, self-maintenance, medication adherence, and depression
	Goldstein et al [45]	RCT (2×2 factorial) + questionnaire	1	60 (4 groups, 15 in each)	69	35	Arm 1: electronic pillbox; arm 2: arm 1 + medication reminder; arm 3: smartphone app; arm 4: arm 3 + medication reminder	Silent App or pillbox (no reminder)	NS: medication adherenceh
	Vuorinen et al [46]	RCT + questionnaire and interview	6	94 (47; 47)	58	17	Telemonitoring via app: monitoring (manual input) weight, BP, HR, and symptoms; automated feedback according to personal targets	Standard care	Between-groups: increase in the use of nurse resources, unplanned clinic visits (both *P*<.001), medication change (increase in *P*=.042; decrease in *P*=.026). NS: heart failure hospital daysh, ED visits, mortality, heart transplant, physiological parameters, and self-care behaviorn
	Seto et al [47]	RCT	6	100 (50; 50)	54	21	Telemonitoring via app + devices (weight and BP): monitoring symptoms; automated feedback; reminders for daily readings; graphical displays	Standard care	Between-groups: increase in self-maintenance (*P*=.03)h,i and quality of life (*P*=.05)g,h; increase in clinic visits NS: self-confidence, self-management, brain natriuretic peptideh, left ventricular ejection fractionh, NYHAh, hospital days, readmissions, mortality, and ED visits
**QE^r^ studies**									
	Heiney et al [48]	QE (1 arm)+questionnaire	1	12	NR^s^	42	App: monitoring (manual input) weight and symptoms; automated feedback; graphical displays; education	None	NS: quality of lifet and self-carei
	Guo et al [49]	QE (1 arm) + intervein + questionnaire	4	66	69	48	Telemonitoring via app + devices (weight, BP, and HR): monitoring symptoms + medication; EMR viewing; graphical displays; remote consultations, clinician communication; visit reminders	None	Increase in consumption of low salt, fat, sugar diet (*P*=.046), fruits, vegetables (*P*=.02); increase in monitoring BP and weight (*P*<.001; *P*=.002); increase in medication adherence (*P*=.006); 60% used the app >1 time/week
	Park et al [50]	QE (1 arm)	1	58	62	33	Telemonitoring via 2 apps + devices (weight and BP): monitoring symptoms and patient-reported outcomes; education; reminders; alerts	None	Readmission rate after intervention: 10% (vs 25% national rates and 23% hospital rate)
	Ware et al [51]	QE (1 arm) + questionnaire + interview	12	232; interview: 24	58; interview: 59	21; interview: 29	Telemonitoring via app + devices (weight, BP, and HR): monitoring symptoms; automated feedback; graphical displays; reminders	None	Overall adherence (days when 4 readings taken/days enrolled): 73.6%. Adherence first month 81.2%; 12 months: 63.1% Age predicted better adherence (*P*=.04)
	Foster [52]	QE (1 arm) + open-ended questionnaire	0.5	10	65	40	App: monitoring (manually) weight, BP, HR, and symptoms; automated feedback; medication reminders; education	None	Increase in self-confidence (*P*=.04)i NS: self-maintenance, self-management, and symptom awareness
	Suthipong [53]	QE (2 arms not randomized)	3	120 (60; 60)	NR	28	App: monitoring (manually) weight, BP, symptoms, and liquid intake; automated feedback; medication adjustments; education; social support; clinician communication	Standard care	Between-groups: decrease in readmission rates (*P*=.04) and pitting edema (*P*<.001); increase in 6-minute walking test (*P*=.01). NS: BP
	Alnosayan et al [54]	QE (1 arm) + interview + questionnaire	6	8	62	38	Telemonitoring via app + devices (weight, BP, and glucose): monitoring symptoms; reminders; education; graphical displays	None	Good usability NS: quality of lifeg
	Radhakrishna et al [55]	QE (1 arm) + questionnaire	1	19	NR	11	Game for tablet: education (quiz and rewards); reminders and tips on self-management	None	Usability: 100% found it easy and enjoyable; increase in heart failure knowledge (*P*=.007)l NS: self-care behaviori

^a^Table is presented in the following order: RCTs first, then quasi-experimental studies, in chronological order of year of publication;

^b^Qualitative findings are included in the *Results* section.

^c^RCT: randomized controlled trial.

^d^BP: blood pressure.

^e^HR: heart rate.

^f^NS: nonstatistically significant.

^g^Measured with the validated questionnaire Minnesota Living with Heart Failure Questionnaire [67].

^h^Indicates primary outcomes.

^i^Measured with the validated questionnaire Self-Care of Heart Failure Index, which measures three subcomponents: self-management, self-confidence, and self-maintenance [68].

^j^Measured with the validated questionnaire EuroQol–5 Dimensions.

^k^ED: emergency department.

^l^Measured with the validated questionnaire Atlanta Heart Failure Knowledge Test [69].

^m^Measured with the validated questionnaire Kansas City Cardiomyopathy Questionnaire score.

^n^Measured with the European Heart Failure Self-Care Behavior Scale.

^o^EMR: electronic medical record.

^p^NYHA: New York Heart Association functional classification.

^q^Measured with the validated questionnaire Dutch Heart Failure Knowledge Scale.

^r^QE: quasi-experimental.

^s^NR: not reported.

^t^Measured with the validated questionnaire Health-Related Quality of Life Scale 14.

**Table 2:** Characteristics of qualitative studies.

First author and country	Methods	Sample size	Age (years), mean	Women, n (%)	Length of app use	Intervention
Schmaderer, United States [56]	Interviews	10	55.8	6 (60)	12 weeks	Same as Schmaderer [39] (Table 1)
Woods, Australia [57]	Questionnaire + interview	6	69	0 (0)	14 days	Smartphone app: monitoring weight, BP^a^, HR^b^, fluid intake, exercise, diet, medication, well-being, and symptoms; graphical display of data; plan setting; reminders and alerts; medical documentation repository, appointments, and care team contacts
Foster, United States [63]	Questionnaires + open-ended questions	10	65	4 (40)	2 weeks	Same as Foster [52] (Table 1)
Portz, United States [62]	Questionnaire + open-ended questions	30	66	18 (60)	NR^c^	Tablet app: monitoring weight and symptoms
Sebern, United States [61]	Focus group + open and closed ended questions	Patients: 4; caregivers: 4; clinicians: 7	Patients: 74; caregivers: 72; clinicians: 34	Patients: 1 (25); caregivers: 3 (75); clinicians: 6 (87)	NR	Tablet app: psychosocial intervention for partners (patients + their caregivers) based on share care, composed of communication (patients’ and caregivers’ preferences and values), decision-making and reciprocity; HF^d^ education
Haynes, United States [60]	Interview (+ thinking aloud user observation)	Patients: 5; clinicians: 3	NR	NR	1 hour	Tablet app: monitoring weight, BP, and symptoms; medication tracking and reconciliation; care team contacts; appointment management
Srinivas, United States [58]	Interview + think-aloud user observation + questionnaire	5	61	2 (40)	60-90 minutes	Tablet app: monitoring weight, BP, HR, symptoms, physical activity, diet, and medication; HF education; daily behavior plan; motivational incentives and rewards
Athilingam, United States [64]	Questionnaires + open questions + user observation	Patients: 25; clinicians: 12	Patients: 58; clinicians: NR	Patients: 10 (40); clinicians: NR	1-2 hours	Same as Athilingam [44] (Table 1)
Seto, Canada [59]	Interview	Patients: 22; clinicians: 5	Patients: 57; clinicians: NR	Patients: 4 (18); clinicians: NR	6 months	Same as Seto [47] (Table 1)

^a^BP: blood pressure.

^b^HR: heart rate.

^c^NR: not reported.

^d^HF: heart failure.

### Intervention Characteristics

Across the 23 apps, the app was provided via a smartphone in 17 (74%) [38-54,57,59,63,64] and via a tablet in 6 (26%) interventions [42,55,58,60-62]. In addition to the app, 35% (8/23) of interventions included telemonitoring (ie, remote monitoring), with transfer of data to health care providers [41,42,46,47,49-51,54], and 65% (15/23) were solely focused on self-management support [38-40,43-45,48,52,53,55,57,58,60-62]. Approximately 9% (2/23) of apps provided patient access to electronic medical records [42,49], and 22% (5/23) of apps allowed direct clinician communication [39,40,42,49,53]. Approximately 48% (11/23) of apps involved patient or clinician co-design [38,40,42-44,47,51,55,57,58,60]. For 39% (9/23) of apps [38,40-42,48,51,53,57,58], the authors reported the use of personalization, mostly in the form of feedback to self-monitored measures (Multimedia Appendix 6 [38-65]).

**Figure 2:**
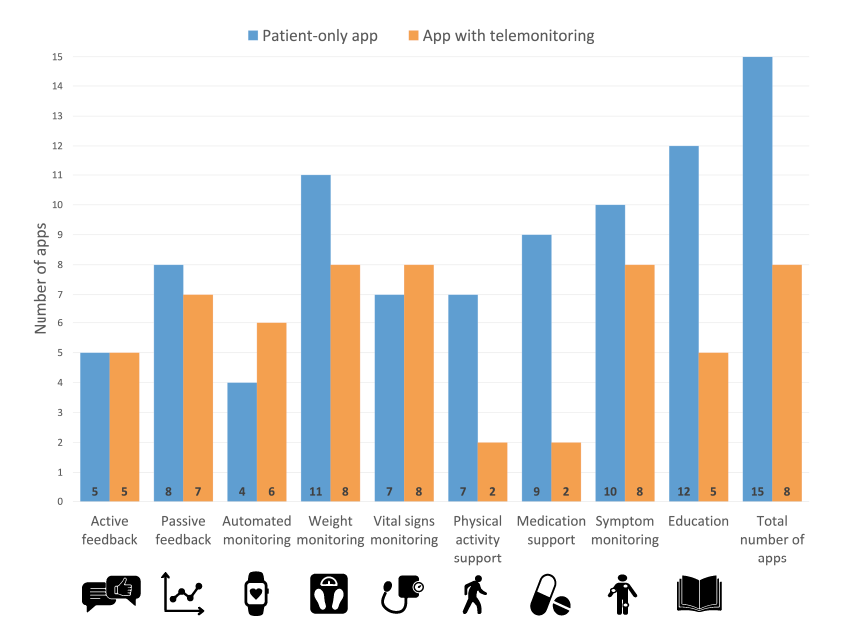
Features present in apps of included studies, grouped by type of app (patient-only app and app with telemonitoring, ie, with transfer of data to health care providers).

The most frequent app features were weight monitoring (19/23, 83%), [38-42,44,46,47,49-54,57-60,62-64], symptom monitoring (18/23, 78%) [40-42,44,46-55,57-60,62-64], and vital signs monitoring (blood pressure and heart rate: 15/23, 65%; Figure 2; Multimedia Appendix 7 [38-55,57-65]) [38,41,42,44,46,47,49-54,57-60,63,64]. Automated monitoring through external wireless devices (eg, weight scale, blood pressure, and heart rate monitor) was present in 43% (10/23) of apps [38-40,42,44,47,49-51,54,59,64]. Of these 10 apps, 6 (60%) were part of a telemonitoring system (ie, the apps were connected to a health care service or clinical provider) [42,47,49-51,54,59]. None of the interventions included implantable cardiac devices. Most apps recommended daily monitoring of symptoms and vital signs, and reminders for monitoring were mentioned in 52% (12/23) of interventions [38-42,44,47,51,53-55,57]. Few studies detailed the format or specifics of symptom monitoring except for 22% (5/23) of interventions [41,46,54,55,62], which allowed for the recording of the presence or absence of specific symptoms, with 20% (1/5) of them based on a validated questionnaire [54] and 60% (3/5) of them also providing symptom severity scales [41,54,62].

The most common BCTs presented in the studies were instructions on how to perform the behavior in 91% (21/23) of interventions [38-55,57-59,61,63,64], followed by self-monitoring of outcomes of behavior in 83% (19/23) [38-42,44,46-54,57-60,62-64], behavioral practice or rehearsal in 78% (18/23) [38-42,44,46-51,53-55,57,59,60,62,64], prompts or cues [38-42,44,45,47-51,53-55,57,59,60,64] and feedback on outcomes of behavior [38-42,44,46-51,53,54,57-59,62,64] in 74% (17/23) of interventions each. Feedback was *active* in 48% (11/23) of apps (ie, the app gave specific instructions to the patient in response to the individual information inputted by them) [40-42,44,46-48,51,53] and *passive* in 65% (15/23) of apps (ie, display of measurements in graphs) [38-40,42,44,47-51,54,57,58,62] (Multimedia Appendix 8 [38-55,57-65]).

### Quantitative Results From Experimental Studies

The 10 included RCTs were small, often underpowered, with main outcomes self-reported, and the results were inconsistent. Approximately 20% (2/10) of RCTs found significant improvements in their primary outcomes: heart failure knowledge [43] and self-care [41]. One of the RCTs [47] reported several primary outcomes, showing improvements in self-care and quality of life. Approximately 40% (4/10) of RCTs did not show significant improvements in their primary outcomes (quality of life [38,39], self-care [38], achieving optimal medical therapy [42], medication adherence [45], and heart failure–related hospital days [46]). Approximately 20% (2/10) of RCTs indicated that their main aim was to assess feasibility [40,44].

Key clinical outcomes in heart failure were seldom reported (ie, mortality [46,47], emergency department visits [39,43,46,47], and hospital readmissions, [39,43,47,50,53]), with only 4% (1/28) of RCTs [39] and 7% (2/28) of quasi-experimental studies [50,53] showing a reduction in readmissions. Approximately 20% (2/10) of RCTs reported higher health care services use in the intervention groups than the control groups, including a higher number of unplanned clinic visits [46,47] and higher use of nurse resources (time and calls) and medication optimization [46].

Other significant improvements were inconsistently reported across experimental studies: heart failure–specific knowledge [40,44,55], self-care [38,52], hospital readmissions [50,53], depression and anxiety measures [38], quality of life [40], systolic blood pressure [42], diet [49], self-monitoring (blood pressure and weight) [49], medication adherence [49], 6-minute walking test [53], and pitting edema [53]. Engagement with the mobile app was reported in 11% (3/28) of studies, 67% (2/3) indicating that less than half of the participants accessed the app daily as recommended by the investigators [40,44] and another showing that 60% of participants used the app more than once a week, as recommended [49].

### User Experience and Qualitative Results From Experimental and Qualitative Studies

#### Overview

User experience was assessed in 68% (19/28) of studies using questionnaires, interviews, and focus groups [40,43-46,48,49,51,52,54,57-65]. The most commonly used questionnaires, apart from those created specifically by study authors, were the System Usability Scale [54,58] and the Unified Theory of Acceptance and Use of Technology questionnaire [51,65].

Of the 28 studies, qualitative analysis to assess acceptability and user perspectives was conducted in 14 (50%) studies (n=8, 57% qualitative-only studies [57-64] and n=6, 21% as part of an experimental study [38,44,46,49,51,52,54]). Common themes were automated self-monitoring and feedback, personalization, communication with clinicians and data sharing and integration, and digital literacy and technical issues.

#### Automated Self-monitoring and Feedback

Most study participants appreciated and noted the importance of automated self-monitoring (particularly through wireless device integration [49,51,54,57,59,60]) and feedback mechanisms with easy-to-understand objective visual displays that could also be tracked by family and friends [46,49,51,54,57,59,64]. They also mentioned that comparing their tracked measures and symptoms with their targets increased goal motivation, symptom awareness, and understanding of the relationship between their lifestyle or behavioral choices and health status, encouraging them to better self-manage their condition [56,59,63].

#### Personalization

Participants in 18% (5/28) of studies noted the need for personalization of the intervention and content provided [51,57,60,62,65] and their preference for more personalization in the ability to report symptoms and needs, which ideally would also generate more relevant feedback [51,57,60]. Specifically, some participants suggested adding a free-writing field [60], additional symptoms [62], and flexibility to input and change information (eg, medication changes) [57]. Personalization of feedback and data displays was also raised, given that some patients found it difficult to interpret longitudinal graphs, and others suggested the ability to increase the size of buttons and text as a desirable feature [57,58]. In addition, the perceived usefulness of the educational content was associated with previous educational level and duration of heart failure, also indicating the importance of personalized educational content [52,55,57]. Reminders for tasks and medication were mentioned as very relevant by most participants in several studies [49,60,62,64].

#### Communication With Clinicians and Data Sharing and Integration

Participants in several studies considered that the app could be an excellent tool for communicating with clinicians and helping with care planning [49,54,56,57,60,61], particularly if it allowed for data sharing and integration with electronic medical records [49,57,60]. Sharing data easily with clinicians, family, and caregivers during emergencies was commonly considered advantageous [49,57,60].

#### Digital Literacy and Technical Issues

Low digital literacy and technical challenges were reported as barriers to using the app in 14% (4/28) of studies [44,49,51,54,57,58,60], and in 4% (1/28) of studies, they were reported as an impassable barrier for older patients without additional technical support [60]. Technical challenges were mentioned as affecting app use and intervention fidelity and were mainly related to difficulties in using the app, such as downloading it, setting reminders, and inputting data [49,57,58,63].

## Discussion

### Principal Findings

In this first systematic review targeting exclusively mobile apps for heart failure self-management, we identified 23 unique apps evaluated in quantitative and qualitative designs, with 8 (35%) being part of telemonitoring systems and connected to health care services. Common features of apps were weight, symptom, and vital signs monitoring and provision of education, medication reminders, and graphical visualization of data. Overall, few had robust efficacy evaluation frameworks—only 10 RCTs involving 717 participants, with ≤6 months of follow-up, substantial heterogeneity in interventions and outcomes, and hence little quantitative evidence to indicate efficacy. Few studies involved patients and clinicians in the design of apps, and few quantified app engagement metrics such as frequency of use during studies. Qualitative studies identified the automation of self-monitoring tasks and feedback, personalization of content and format, communication with clinicians, and data sharing and integration capabilities as key enablers.

### Comparison With Existing Literature

Similar to previous systematic reviews of other digital technologies in heart failure (focused on nonapp mobile technologies, such as SMS text messaging, personal digital assistants, interactive voice response, and phone calls), our findings were mixed, with high heterogeneity and lack of detailed reporting of intervention characteristics [18-27] likely because of poor evaluation frameworks. In these reviews, the interventions did not commonly offer self-management support (eg, education and feedback), merely involving remote monitoring with regular digital transmission of physiological and other disease-related data from the patient’s home to a health care center. In addition, previous nonsystematic reviews seemingly with a focus on apps for heart failure self-management either only assessed the content and quality of commercially available apps [28-30] or broadened their inclusion criteria, including studies where the intervention was some type of mHealth technology but not an app (eg, SMS text messaging) [30]. In contrast, our systematic review is the first to focus exclusively on mobile apps for heart failure self-management (with or without clinician involvement via telemonitoring).

Despite the focus on heart failure self-management, the studies included in this review varied considerably in the types of self-management support features available in the apps. Core components of heart failure self-management programs, as defined in existing guidelines [2,5,70], include education, symptom monitoring, medication support, and physical activity support. Nevertheless, only 26% (6/23) of apps provided all these features [44,52,54,55,57,58], with more apps including features less supported by evidence in regard to their benefits in heart failure [5], such as daily weight monitoring. As a road map for future studies in this area, we encourage researchers and developers to follow the best available evidence [2,5,70] when designing and evaluating heart failure apps for self-management, focusing on features that have been systematically associated with improved outcomes. In addition, better reporting of intervention features is crucial to avoid what has been named as the *black box* of home telemonitoring [20], where the specific effective components of these interventions remain unknown.

Key outcomes in heart failure were seldom assessed in the included studies, hampering a complete evaluation of the impact of heart failure self-management apps. Overall, 1 RCT [39] and 2 quasi-experimental studies showed a significant reduction in readmissions [50,53], corresponding to the evaluation of 1 self-management app with telemonitoring and 2 without telemonitoring. Furthermore, 30% (3/10) of RCTs evaluated health care system use [43,46,47], with 67% (2/3) of them finding a higher number of unplanned clinic visits and medication optimization for participants in telemonitoring programs [46], although without significant changes in mortality, emergency department visits, or hospitalization [46,47]. Higher health care use may reflect earlier actions in the face of signs of worsening heart failure and provide opportunities for medication optimization. Such results may help explain the positive outcomes of telemonitoring interventions [26]. Longer and adequately powered studies measuring key clinical outcomes are needed to fully assess whether the potential benefits of self-management apps outweigh the costs of increased health care use.

Self-reported measures were commonly assessed in experimental studies, including validated questionnaires to measure heart failure knowledge, self-care, and quality of life [67-69]. Heart failure knowledge was significantly improved in 14% (4/28) of studies, all of which involved apps without telemonitoring [40,43,44,55]. Self-care was improved in 14% (4/28) of studies [38,41,47,52], 50% (2/4) of which involved apps with telemonitoring [41,47], and quality of life improved in 7% (2/28) of studies [40,47], 50% (1/2) of which involved telemonitoring [40]. There has been increasing recognition of the importance of including patient-reported outcomes as end points when evaluating interventions, as well as the benefits of collecting them routinely to improve care [71-73]. Digital technologies such as mobile apps can facilitate the capture of patient-reported outcomes, such as symptom status and severity [71], which can then be used by clinicians to guide care. Nevertheless, only one of the apps used a validated questionnaire for symptom monitoring [54]. The potential of mobile apps to collect patient-reported outcomes should be further explored in future studies, given their ability to promote patient-centered care and improve the quality of care for patients.

Overall, the evidence on the use of mobile apps for heart failure self-management is still lagging behind the large body of work supporting mHealth for remote monitoring, where significant reductions in all-cause mortality have been reported [19-22,26,27]. In our review, all included studies focused on supporting heart failure self-management, with 44% (8/18) of experimental studies including a telemonitoring component with clinician involvement [46,47,49-51,54]. Unfortunately, the small number, size, and quality of these studies do not enable us to draw conclusions regarding potential differences in efficacy between these 2 different types of mobile app interventions for heart failure self-management—with or without telemonitoring. Given the demonstrated benefits of self-management interventions more broadly [74] and remote monitoring [18-27], future research should explore the possibility that their combination may result in synergistic effects and higher efficacy in improving heart failure outcomes.

Personalization was valued in the studies included in this review, particularly personally relevant feedback and tailoring of the intervention to different levels of education and digital literacy. These findings are similar to those involving apps for other chronic diseases, showing that enabling customization (eg, editing information and choosing which aspects to track) is among the most appealing features and may enhance the usability, motivation, and engagement with the apps [17,75,76]. Future studies may explore the delivery of core BCTs (self-monitoring, feedback, and instruction on how to perform the behavior) and provide other techniques in a personalized manner, according to patient preferences and self-reported information [77] or based on machine learning algorithms using patient data collected over time (eg, from smartphone sensors or wireless monitoring devices) [78,79].

Limited experience in using technology can be a barrier to using mobile apps and may affect the utility and perceived benefit of mobile apps, as shown by our findings. The lack of confidence in using technology and perceived capability to benefit from it, as well as the workload required to learn how to use an app, are particularly challenging among older patients [80,81]. A study conducted to understand the main facilitators of and barriers to the use of mobile technology among older adults found that the most often mentioned barrier was the lack of knowledge on how to use it, whereas having previous experience of use was a facilitator [82]. However, older patients are willing to learn how to use mHealth technology and feel it may help them improve and maintain self-care behaviors [82,83]. Given that a large population of patients experiencing heart failure involves older adults, future app development needs to take into account specific characteristics of this population to design apps with simple navigation and ease of use [81].

### Strengths and Limitations

This study presents several strengths. The PRISMA protocol was systematically followed. The screening process was pilot tested before its start, and there was good agreement between the independent reviewers. We also included both experimental and qualitative studies, enabling a better understanding of the impact, acceptance, and user preferences regarding mobile apps for heart failure self-management.

Some limitations should be considered in the interpretation of our results. First, given the heterogeneity between interventions and the small number of RCTs, a meta-analysis was not conducted. Second, the heterogeneity of study designs, sample sizes, follow-ups, interventions, and outcome measures among the experimental studies did not allow for consistent conclusions on the effectiveness of mobile apps in heart failure. Third, some studies in this review included analysis of adherence, acceptability, or usability of their interventions; however, although favorable trends were reported, the different measures and definitions used hindered reliable conclusions. Fourth, the socioeconomic and clinical characteristics of participants were rarely reported in the included studies; however, when reported, they suggested a high educational level and mild to moderate disease severity, potentially limiting the generalizability of the findings. Finally, the nature of this kind of research hampers the proper elucidation of the sociotechnical aspects of the interventions, which should be further evaluated in future studies (eg, using realist review methods).

### Implications for Research, Clinical Practice, and Policy

Despite growing interest in the use of mobile apps for heart failure self-management, critical gaps remain in their design and evaluation, with lack of patient and clinician involvement and lack of robust evaluation to determine the populations that may benefit the most. Given the importance of patient preference and engagement in the successful delivery of heart failure interventions [26,27], co-design processes involving clinicians and patients and process evaluations assessing engagement and acceptability of the interventions are likely to improve intervention quality and consistency. Future studies should follow existing evidence in designing apps with features most likely to improve key patient-reported and clinical outcomes, adhering to recommendations derived from this study (Textbox 1). In addition, they should explore the efficacy and cost-effectiveness of mobile apps for heart failure self-management with and without a telemonitoring component. It is possible that self-management interventions without telemonitoring may be sufficient to improve outcomes in the early stages of disease in patients with a low risk of premature morbidity and mortality.

**Recommendations for researchers and developers regarding apps for heart failure self-management.**

**Recommendations for researchers and developers**

Researchers and developers, when designing and evaluating apps, should consider the following:

- Follow the best available evidence
- Align with clinical guidelines
- Use co-design and pilot-testing to optimize products
- Enable automated self-monitoring and feedback, personalization, communication with clinicians, and data sharing and integration
- Report on specific functionalities and features of the apps
- Evaluate effectiveness on relevant outcomes to heart failure patients; for example, clinical outcomes, health service use, and clinical measures
- Report on adverse events or inadvertent effects; for example, increased health care use
- Patient-reported outcomes, including self-care and experiences, are also important; however, consider the ability to compare such measures among studies

Research is needed to better understand how these interventions can be implemented in the real world and integrated into existing models of care, such as collaborative care models involving shared care between heart failure nurses, general practitioners, and cardiologists [84-86]. Integrating these interventions into such services may increase their benefits and leverage partnerships between patients and clinicians, possibly leading to a more seamless implementation in practice. Perhaps a future model of care for heart failure patients can involve using mobile technology to improve patients’ confidence and ability to manage their condition with greater autonomy, coupled with telemonitoring with clinician support for higher-risk patients.

### Conclusions

This systematic review showed that research on the use of apps in heart failure self-management is still at an early stage, with limited evidence supporting its efficacy. RCTs are needed to fully ascertain the impact of these interventions. Future research should encompass greater involvement of end users and comprehensively measure patient engagement with the intervention.

## Appendix

Multimedia Appendix 1 Search strategy.

Multimedia Appendix 2 PRISMA (Preferred Reporting Items for Systematic Reviews and Meta-Analyses) checklist.

Multimedia Appendix 3 List of articles excluded after full-text review for not meeting inclusion criteria regarding the intervention, outcome, or unavailability of full-text.

Multimedia Appendix 4 Patients’ clinical and socioeconomic characteristics.

Multimedia Appendix 5 Quality assessment of included studies.

Multimedia Appendix 6 Presence of personalization in included studies.

Multimedia Appendix 7 Intervention features of included articles.

Multimedia Appendix 8 Behavior change techniques present in the interventions of included articles.

## Abbreviations

BCT: behavior change technique
mHealth: mobile health
PRISMA: Preferred Reporting Items for Systematic Reviews and Meta-Analyses
PROSPERO: International Prospective Register of Systematic Reviews
