# Supplementary material for: The Use of Mobile Apps for Heart Failure Self-management: Systematic Review of Experimental and Qualitative Studies
Source: JMIR Cardio. 2022 Mar 31;6(1):e33839. doi: 10.2196/33839 (PMC9015755; doi:10.2196/33839)
Supplement: Multimedia Appendix 1 [file cardio_v6i1e33839_app1.docx]

## Multimedia Appendix 1: Search strategy

The reference lists of relevant articles and grey literature were also screened to ensure all eligible studies were captured. The search was limited from 2008 onwards since most app stores were launched in that year. No language limits were applied.

Electronic bibliographic databases and platforms

Date: 24 Oct 2019

**1.1. Search strategy for PubMed**

URL: Macquarie University Library

Limits: 2008 - current

**#1** ("cell phone"[MeSH Terms] OR "cell phone"[All Fields] OR "smartphone"[MeSH Terms] OR "smartphone"[All Fields] OR "mobile phone"[All Fields] OR "mobile applications"[MeSH Terms] OR "mobile applications"[All Fields] OR "mobile application"[All Fields] OR "telemedicine"[MeSH Terms] OR "telemedicine"[All Fields])

**#2** ("heart failure"[MeSH Terms] OR "heart failure"[All Fields])

**#3** ("self-management"[MeSH Terms] OR "self-management"[All Fields] OR "self management"[All Fields] OR "self care"[MeSH Terms] OR "self care"[All Fields] OR ("self"[All Fields] AND “monitoring”[All Fields]) OR “self-monitoring”[All Fields] OR "monitoring, physiologic"[MeSH Terms] OR ("monitoring"[All Fields] AND "physiologic"[All Fields]) OR "physiologic monitoring"[All Fields] OR ("physiologic"[All Fields] AND "monitoring"[All Fields]) OR ("patient"[All Fields] AND "monitoring"[All Fields]) OR "patient monitoring"[All Fields] OR "patient participation"[MeSH Terms] OR "patient participation"[All Fields] OR ("patient"[All Fields] AND "activation"[All Fields]) OR "patient activation"[All Fields])

**#4** **#1 AND #2 AND #3**

**1.2. Search strategy for EMBASE**

URL: Macquarie University Library (via OVID interface)

Limits: 2008 - current

**#1** Mobile phone/ or smartphone/ or mobile application/ or mobile health application/ or (“app” or “apps” or “application” or “applications”).mp

**#2** Heart failure/ or congestive heart failure/ or diastolic dysfunction/ or systolic dysfunction/ or (“heart failure*” or “congestive heart failure*”).mp

**#3** Self care/ or self monitoring/ or monitoring/ or physiologic monitoring/ or (“self-management*” or “self-care*” or “self-monitoring*” or “patient activation” or “physiological monitoring*” or “tracking*”).mp

**#4 #1 AND #2 AND #3**

**1.3. Search strategy for CINAHL**

URL: Macquarie University Library (via EBSCO Publishing)

Limits: 2008 - current

**#1** (MH “heart failure”) or “heart failure*”

**#2** (MH "Smartphone") or (MH "Mobile Applications") or (MH "Cellular Phone") or “smartphone*” or “mobile phone*” or “cell phone*” or “mobile health*” or “app” or “application” or “apps” or “applications”

**#3** (MH "Self-Management") or (MH "Self Medication") or (MH "Self Administration") or (MH "Self Care") or (MH "Monitoring, Physiologic") or “self-management*” or “self-care*” or “self-monitoring*” or “patient activation” or "physiological monitoring" or “tracking*”

**#4** **#1 AND #2 AND #3**

**1.4. Search strategy for PsycINFO**

URL: Macquarie University Library (via OVID interface)

Limits: 2008 - current

**#1** Mobile phones/ or mobile devices/ or smartphones/ or mobile applications/or mobile health/ or mobile technology/ or (“mobile phone*” or “smartphone*” or “cell phone*” or “app*” or “application*”).mp

**#2** (“heart failure*” or “congestive heart failure*” or “ventricular dysfunction*”).mp

**#3** Self-management/ or self-monitoring/ or self-care skills/ or monitoring/ or tracking/ or (“self-management*” or “self-monitoring*” or “self-care*” or “patient activation” or “physiological monitoring*”.mp

**#4** **#1 AND #2 AND #3**
