# Supplementary figures and images for: The Use of Mobile Apps for Heart Failure Self-management: Systematic Review of Experimental and Qualitative Studies
Source: JMIR Cardio. 2022 Mar 31;6(1):e33839. doi: 10.2196/33839 (PMC9015755; doi:10.2196/33839)

## Multimedia appendix 2: PRISMA 2020 checklist and PRISMA 2020 for abstracts checklist
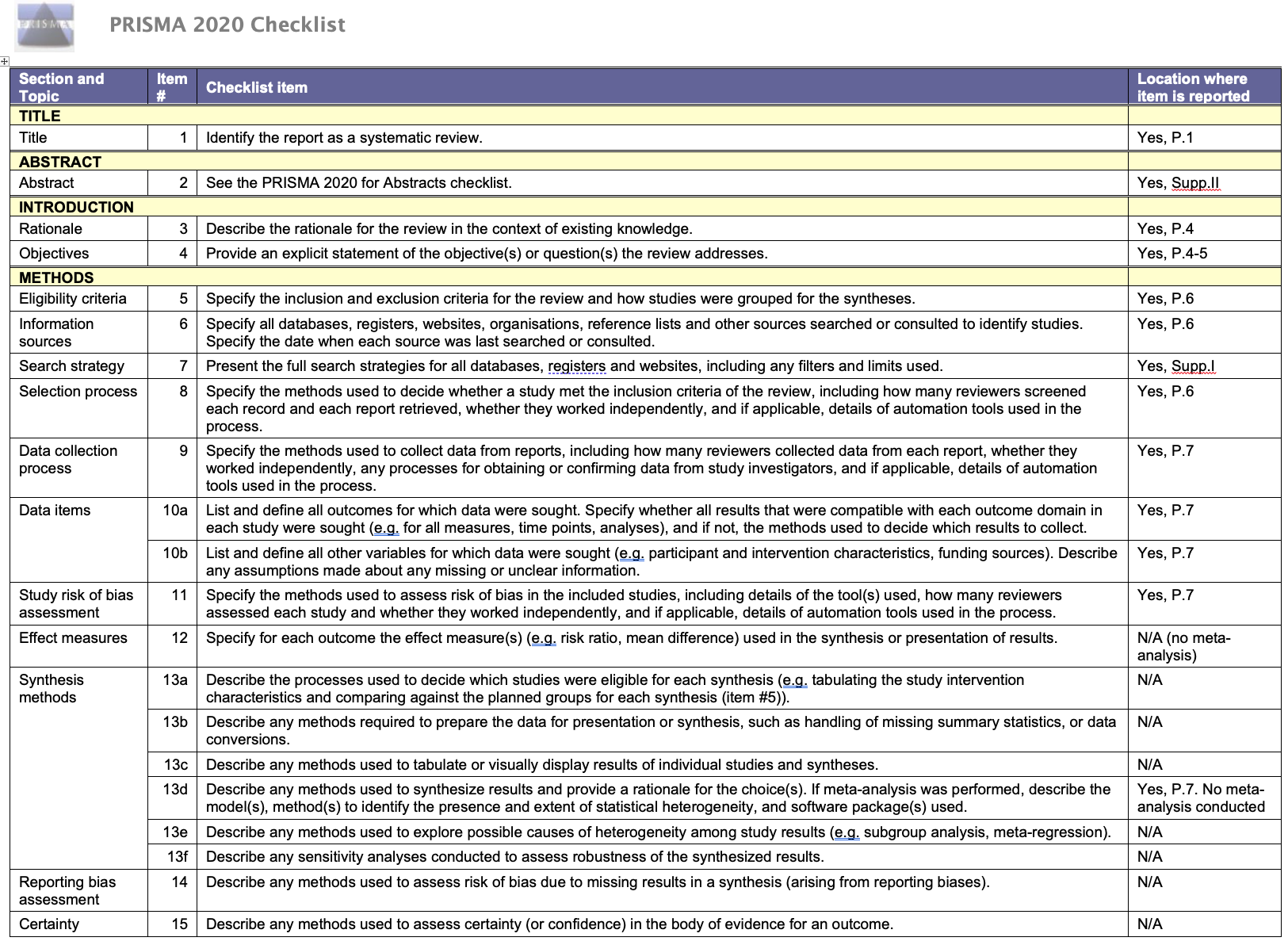


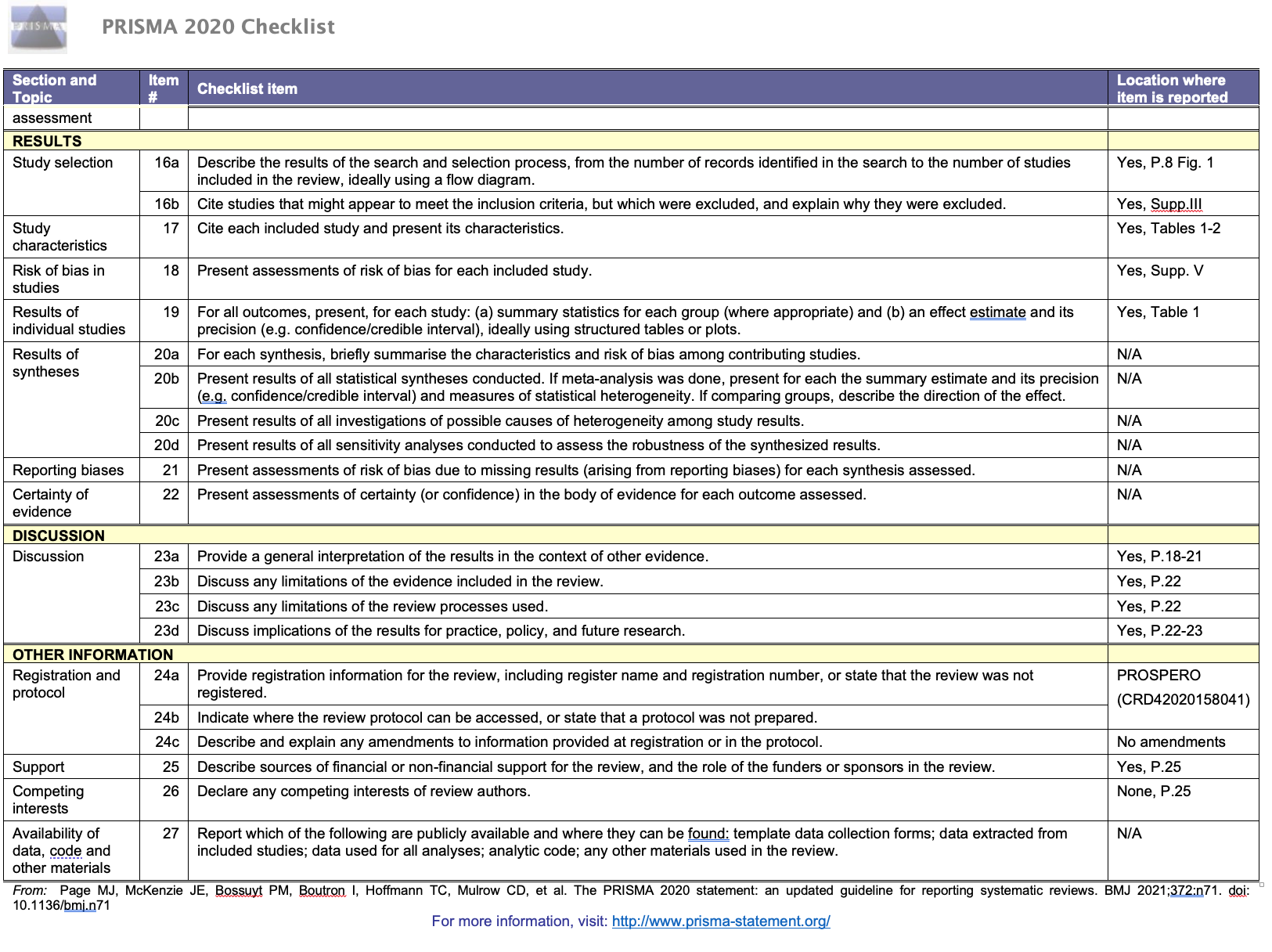


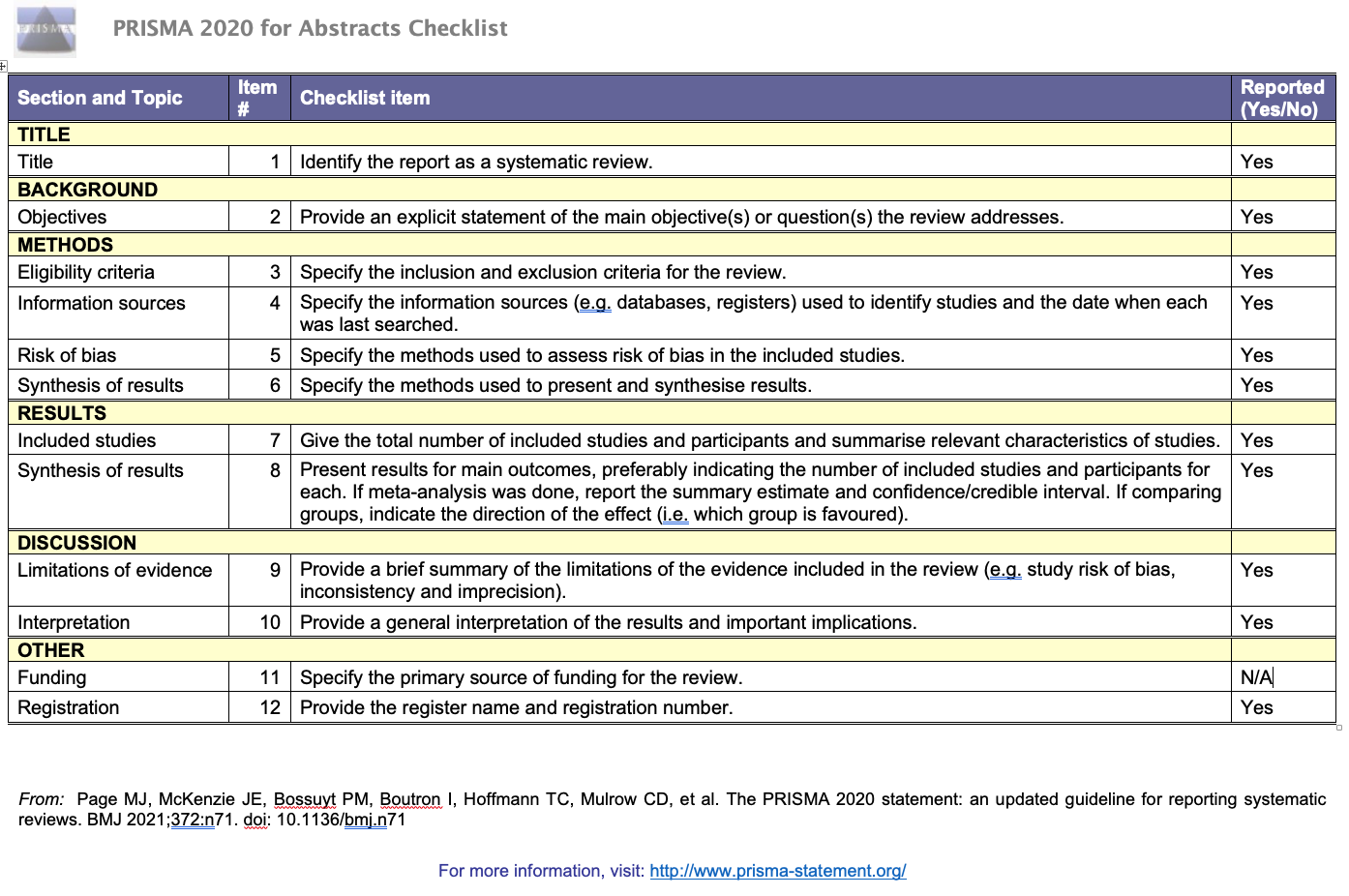

Supplement: Multimedia Appendix 2 [file cardio_v6i1e33839_app2.docx]
