# Supplementary material for: The Use of Mobile Apps for Heart Failure Self-management: Systematic Review of Experimental and Qualitative Studies
Source: JMIR Cardio. 2022 Mar 31;6(1):e33839. doi: 10.2196/33839 (PMC9015755; doi:10.2196/33839)
Supplement: Multimedia Appendix 3 [file cardio_v6i1e33839_app3.docx]

## Multimedia appendix 3: List of articles excluded after full-text review for not meeting inclusion criteria regarding the intervention, outcome, or unavailability of full-text

**Intervention**:

Thakur RD. Feasibility study of the health empowerment intervention to evaluate the effect on self- management, functional health, and well-being in older adults with heart failure. Dissertation Abstracts International: Section B: The Sciences and Engineering. 2018;78(8-B(E)):No Pagination Specified.

Jaana M, Sherrard H, Paré G. A prospective evaluation of telemonitoring use by seniors with chronic heart failure: Adoption, self-care, and empowerment. 2019;25(4):1800-14.

Dang S, Karanam C, Gomez-Orozco C, Gomez-Marin O. Mobile Phone Intervention for Heart Failure in a Minority Urban County Hospital Population: Usability and Patient Perspectives.23(7):544-54.

Dang S, Karanam C, Gomez-Marin O. Outcomes of a Mobile Phone Intervention for Heart Failure in a Minority County Hospital Population. Telemed J E Health. 2017;23(6):473-84.

Werhahn SM, Dathe H, Rottmann T, Franke T, Vahdat D, Hasenfuss G, et al. Designing meaningful outcome parameters using mobile technology: a new mobile application for telemonitoring of patients with heart failure. ESC heart failure. 2019;6(3):516-25.

Rosen D, McCall JD, Primack BA. Telehealth Protocol to Prevent Readmission Among High-Risk Patients With Congestive Heart Failure. American Journal of Medicine. 2017;130(11):1326-30.

Melin M, Hagglund E, Ullman B, Persson H, Hagerman I. Effects of a tablet computer on self-care, quality of life, and knowledge: A randomized clinical trial. Journal of Cardiovascular Nursing. 2018;33(4):336-43.

**Study design**:

Cano Martin JA, Martinez-Perez B, de la Torre-Diez I, Lopez-Coronado M. Economic impact assessment from the use of a mobile app for the self-management of heart diseases by patients with heart failure in a Spanish region. J Med Syst. 2014;38(9):96.

**Outcome**:

Triantafyllidis A, Velardo C, Chantler T, Shah SA, Paton C, Khorshidi R, et al. A personalised mobile-based home monitoring system for heart failure: The SUPPORT-HF Study. International Journal of Medical Informatics. 2015;84(10):743-53.

**No full-text available**:

Lopatin Y, Grebennikova A, Stoliarov A, Jaarsma T. Will an interactive smartphone application improve self-care behavior and quality of life in patients with heart failure? European Heart Journal. 2017;38 (Supplement 1):300-1.

Karanam C, Dayanand S, Dang S, Cobian S, Gomez-Marin O, Mallon S, et al. Outcomes from a mobile-phone study for heart failure in an ethnically diverse County Hospital. Journal of the American Geriatrics Society. 2012;4):S221.

Linda Houston-Feenstra L, Dysinger W, Kagoda M, Chatterjee S, Alnosayan N, Alluhaidan A. Transition to home in heart failure: Theres an app for that. European Journal of Preventive Cardiology. 2015;1):S166.

Kapoor A, Koul R, Singh A, Chir G. Evaluation, Assessment and Development of Artificial Intelligence Based Heart Failure Platform- a Precision Based Approach. Canadian Journal of Cardiology. 2019;35 (10 Supplement):S47-S8.

Grebennikova A, Stoliarov A, Jaarsma T, Yury Lopatin YM. The interactive smartphone application for the improvement of self-care in patients with heart failure. European Journal of Heart Failure. 2017;19 (Supplement 1):178.

Grebennikova AA, Stoliarov AY, Lopatin YM. The use of platform for remote monitoring on the base of mobile app for improving self-care in patients with chronic heart failure. [Russian]. Kardiologiia. 2017;57(S4):11-8.

Georgios Zisis G, Carrington MJ, Ball J, Marwick T. Heart failure digital coach: Pilot findings of an avatar style application to improve symptoms, self-care and knowledge of heart failure. European Journal of Heart Failure. 2019;21 (Supplement 1):440-1.

Du H, Burdeniuk C, Kelman S, Nolan P, Barry T, Clark RA. Development and feasibility testing of an avatar-based education application for improving heart failure patients' knowledge and self-care behaviours. European Journal of Heart Failure. 2019;21 (Supplement 1):179.

Dorsch MP, Farris KB, Hummel SL, Koelling TM. A Patient-Centered Mobile Intervention to Promote Self-Management and Improve Patient Outcomes in Chronic Heart Failure: The ManageHF Trial. Journal of Cardiac Failure. 2019;25 (8 Supplement):S104.

Baik D, Creber RM. Using m-Health to measure symptoms and evaluate heart status in patients with heart failure. Circulation Conference. 2018;138(Supplement 1).

Athilingam P, Osorio RE, Kaplan H, Oliver D, O'Neachtain T, Rogal PJ. Embedding Patient Education in Mobile Platform for Patients With Heart Failure: Theory-Based Development and Beta Testing. Comput Inform Nurs. 2016;34(2):92-8.

Wonggom P, Du H, Nolan P, Burdeniuk C, Kelman S, Barry T, et al. Development of an Avatar-Based Education Application for Improving Knowledge and Self-Care behaviours in Heart Failure: A Feasibility Study. Heart, Lung & Circulation. 2019;28:S342-S3.

Wolf A, Olsson LE, Swedberg K, Ekman I. Use of smartphones in person-centred e-health diaries in patients above 75 years. European Journal of Heart Failure, Supplement. 2012;1):S1.

Otobo E, Atreja A, Freeman R, Rogers J, Fasihuddin F, Deorocki A, et al. Use of electronic patient reported outcomes and automated devices for heart failure disease management. Journal of the American College of Cardiology Conference: 67th Annual Scientific Session of the American College of Cardiology and i2 Summit: Innovation in Intervention, ACC. 2018;71(11 Supplement 1).

Clark RA, Fredericks B, Adams M, Atherton J, Howie-Esquivel J, Dracup K, et al. Addressing health literacy and cultural teaching issues in australian indigenous and non-indigenous heart failure patients using avatars: Technology development and pilot testing. Journal of Cardiac Failure. 2014;1):S115.

Cichosz SL, Udsen FW, Hejlesen O. The impact of telehealth care on health-related quality of life of patients with heart failure: Results from the Danish TeleCare North heart failure trial. J Telemed Telecare. 2019:1357633x19832713.

Vathsangam H, Qiao S, Berkley J, Adesanya A, Sukhatme G, Grazette L, et al. Smartphone delivered cardiac rehab for heart failure management: A feasibility study. Circulation Conference: American Heart Association's. 2015;132(SUPPL. 3).

Foster M. A Mobile Application for Patients With Heart Failure: Theory- and Evidence-Based Design and Testing. CIN: Computers, Informatics, Nursing. 2018;36(11):540-9.
