# Supplementary material for: The Use of Mobile Apps for Heart Failure Self-management: Systematic Review of Experimental and Qualitative Studies
Source: JMIR Cardio. 2022 Mar 31;6(1):e33839. doi: 10.2196/33839 (PMC9015755; doi:10.2196/33839)
Supplement: Multimedia Appendix 4 [file cardio_v6i1e33839_app4.docx]

## Multimedia appendix 4: Patients’ clinical and socioeconomic characteristics

| First author, year | NYHA classification (%) | Mean LVEF (%) | Etiology (%) | HF duration | Race or Ethnicity (%) | Educational level (%) |
| --- | --- | --- | --- | --- | --- | --- |
| **Experimental studies** | | | | | | |
| Clays, 2021 | II: 87  III: 13 | 32 | Ischemic: 87 | - | - | - |
| Schmaderer, 2021 a | II: 24.3  III: 43.2  IV: 21.6  NR: 10.8 | 33.7 | - | - | White: 55  Black: 34  Other: 10 | ≤HS: 50  ≥HS: 50 |
| Wei, 2021 | I: 14  II: 32  III: 11  IV: 4 | Intervention: 47.4  Control: 40.9 | - | - | White: 68  Other: 14 | - |
| Yanicelli, 2021 | I: 12.5  II: 40  III: 20  IV: 2.5 | - | - | - | - | <HS: 43  HS: 18 |
| Rahimi, 2020 | I: 32; 33 (I;C)  II: 24; 32  III: 37; 26  IV:8; 10 | 32.9 | - | - | - | - |
| Wonggom, 2020 | I: 50  II: 42  III:8.3 | - | Dilated: 42  Idiopathic: 28  Ischemic: 14  Others: 17 | >5years: 47  <1year: 28  1-5 years: 25 | - | ≤HS: 25  Technical: 61  Degree: 14 |
| Athilingam P, 2017 | I: 6  II: 61  III: 33 | 28 | Non-ischemic: 89 | 67% >1year | White: 50  Black: 33.3  Hyspanic: 17 | <HS: 6  HS: 33  ≥HS: 61 |
| Goldstein CM, 2014 | II and III* | <40* | - | ≥ 3 months* | White: 81 | <HS: 7  HS: 31  Technical: 12  Some college: 31  Bachelor: 14  Master: 5 |
| Vuorinen AL, 2014  (IG) | II: 40  III: 58  IV: 2 | 27 | - | >6 months* | - | - |
| Vuorinen AL, 2014  (CG) | II: 36  III: 60  IV: 4 | 29 | - | >6 months* | - | - |
| Seto E, 2012  (IG) | II: 42  II-III: 12  III: 42  IV: 4 | 27 | Ischemic: 40  Idiopathic: 44  Others: 16 | Average 4.8 years | White: 78  Black: 10  Asian: 4  Other: 8 | <HS: 2  HS: 24  College: 66 |
| Seto E, 2012  (CG) | II: 44  II-III: 10  III: 42  IV: 4 | 27 | Ischemic: 26  Idiopathic: 58  Others: 16 | Average 3.5 years | White: 66  Black: 8  Asian: 10  Other: 16 | <HS: 12  HS: 26  College: 56 |
| Heiney, 2020 | - | - | - | <1y: 58  2-8y: 25  >8: 17 | Black: 100 | <HS: 17  HS: 58  >HS: 25 |
| Guo X, 2019 | I: 17  II: 48  III: 35  IV: 0 | ≤45%* # | - | ≥3 months* | - | <HS: 77  ≥HS: 23 |
| Park C, 2019 | - | - | - | - | White: 31  Black: 24  Other: 45 | - |
| Ware P, 2019 | ≤II: 49  II-III: 21  ≥III: 30 | 32 | - | - | White: 66  Black: 8  Asian: 12.1  Other: 13.8 | <HS: 7.5  HS:19.5  College: 73 |
| Foster M, 2018 | II: 70 | - | - | 1 month to 9 years (mean 2.4) | White: 90 | ≥ Some college: 90 |
| Suthipong C, 2018 | II* | - | Ischemic: 61.7  Valvar: 33  Others: 5.8 | ≥6 months* | - | <HS: 58.4  HS: 22.5  Bachelor: 10  Master: 8.3  PhD: 0.8 |
| Alnosayan N, 2017 | III or IV* | 45-70* | - | ≥6 months* | - | - |
| Radhakrishnan K, 2016 (Functionality) | - | - | - | <2y:1  2-5y: 7  6-10y: 0  >10y: 11 | White: 84.2 | <HS: 7  HS: 31  Technical: 12  Some college: 31  Bachelor: 14  Master: 5 |
| **Qualitative studies** | | | | | | |
| Schmaderer, 2020 b | III: 50  IV: 50 | <50%: 90  ≥50%: 10 | - | - | White: 40  Other: 60 | Mean 13.5 y |
| Woods L, 2019 | - | - | - | - | - | - |
| Foster M, 2018 | - | - | - | Average 2.4 years | White: 90  Other: 10 | ≤ HS: 10  ≥2year in college: 90 |
| Portz JD, 2018 | - | - | - | Average 8.8 years | White: 33.3  Black: 63.3  Other: 3.3 | - |
| Sebern MD, 2018 | - | - | - | Average 19 years | White: 100 | <HS: 25  HS: 25  College: 25  Post college: 25 |
| Haynes SC, 2017 | - | - | - | - | - | - |
| Srinivas P, 2017 | Mild, moderate, or severe functional status* | - | - | - | White non-Hispanic: 74 | HS: 34  <HS: 15  College: 51 |
| Athilingam P, 2016 | I: 4  II: 64  III: 28  IV: 4 | - | Non-ischemic 68 | ≤3 y: 48%  ≥4 y: 52% | White: 40  Black: 36  Hyspanic: 20  Other: 4 | ≤ HS: 36 |
| Seto E, 2012 | - | <40* | - | - | - | - |

*condition in the inclusion criteria; #LVEF>45% accepted if HF caused by atrial fibrillation, valvular heart disease, or hypertrophic cardiomyopathy

Abbreviations: AF: atrial fibrillation; C: Control; HF: heart failure; HS: high school; I: Intervention; LVEF: left ventricular ejection fraction; NYHA: New York Heart Association functional classification; y: years
