# Supplementary material for: The Use of Mobile Apps for Heart Failure Self-management: Systematic Review of Experimental and Qualitative Studies
Source: JMIR Cardio. 2022 Mar 31;6(1):e33839. doi: 10.2196/33839 (PMC9015755; doi:10.2196/33839)
Supplement: Multimedia Appendix 5 [file cardio_v6i1e33839_app5.docx]

## Multimedia appendix 5: Quality assessment of included studies

**Quality Rating of individual randomized controlled trials using Cochrane’s risk of bias tool^a^**

| Author, year, country | Random sequence allocation | Allocation concealment | Blinding of participants and personnel | Blinding of outcome assessment | Incomplete outcome data | Selective reporting |
| --- | --- | --- | --- | --- | --- | --- |
| Clays, 2021 | ? | ? | - | - | + | + |
| Schmaderer, 2021a | ? | ? | - | - | + | ? |
| Wei, 2021 | ? | ? | - | - | + | - |
| Yanicelli, 2021 | + | ? | - | - | + | + |
| Rahimi, 2020 | + | + | + | ? | + | ? |
| Wonggom, 2020 | + | + | - | - | + | + |
| Vuorinen, 2014 | ? | ? | - | + | + | + |
| Seto, 2012 | + | + | - | + | + | + |
| Athilingam, 2017 | ? | ? | - | - | + | ? |
| Goldstein, 2014 | + | - | - | + | + | + |

+: Low risk of bias; -: High risk of bias; ?: Unclear risk of bias

^a^Higgins JP and Green S. *Cochrane handbook for systematic reviews of interventions: Cochrane Book Series*. 2008, p.1-649.

**Quality Rating of individual non-randomized studies using the ‘Risk Of Bias In Non-randomised Studies - of Interventions’ (ROBINS-I) tool^b^**

| **Assessment of risk of bias using the Risk Of Bias In Non-randomized Studies – of Interventions (ROBINS-I) assessment tool** | | | | | | | | |
| --- | --- | --- | --- | --- | --- | --- | --- | --- |
| **Risk of bias pre-intervention and at-intervention domains** | | | | **Risk of bias post-intervention domains** | | | |  |
| **Study** | **Bias due to confounding** | **Bias in selection of participants into the study** | **Bias in classification of interventions** | **Bias due to deviations from intended intervention** | **Bias due to missing data** | **Bias in measurement of outcomes** | **Bias in selection of the reported result** | **Overall Assessment of bias** |
| **Experimental studies** | | | | | | | | |
| Heiney, 2020 | Critical | Serious | Low | Serious | Low | Critical | No information | Serious |
| Guo, 2019 | Critical | Low | Low | Low | Low | Critical | No information | Serious |
| Park, 2019 | Critical | Low | Low | Serious | Low | Critical | No information | Serious |
| Ware, 2019 | Critical | Low | Low | Low | Low | Critical | No information | Serious |
| Foster, 2018 | Critical | Serious | Low | Critical | Low | Critical | No information | Critical |
| Suthipong, 2018 | Critical | Low | Low | No information | Low | Critical | No information | Critical |
| Alnosayan, 2017 | Critical | Serious | Low | Critical | Low | Critical | No information | Critical |
| Radhakrishna, 2016 | Critical | Serious | Low | No information | Low | Critical | No information | Critical |

Key: Low risk of bias, Moderate risk of bias, Serious risk of bias, Critical risk of bias, No information

^b^Sterne JA, Hernán MA, Reeves BC, et al. ROBINS-I: a tool for assessing risk of bias in non-randomised studies of interventions. *BMJ* 2016;**355**:i4919 doi: 10.1136/bmj.i491
