# Supplementary material for: The Use of Mobile Apps for Heart Failure Self-management: Systematic Review of Experimental and Qualitative Studies
Source: JMIR Cardio. 2022 Mar 31;6(1):e33839. doi: 10.2196/33839 (PMC9015755; doi:10.2196/33839)
Supplement: Multimedia Appendix 6 [file cardio_v6i1e33839_app6.docx]

## Multimedia appendix 6: Presence of personalization in included studies

| **Author, year** | **Intervention** | **Personalization - quotes from the article mentioning personalization or synonyms (e.g. customization, tailoring)** |
| --- | --- | --- |
| **Experimental studies** | | |
| Clays, 2021 | App | “comprises personalized systems for nutrition advice, self-monitoring, medication intake and disease education; personalisation to the individual patient’s clinical and psychological profile” |
| Schmaderer, 2021 a | App | NR |
| Wei, 2021 | App | “Personalized coaching” |
| Yanicelli, 2021 | App + TM | “Personalized alert system” |
| Rahimi, 2020 | App + TM | “Personalised feedback” |
| Wonggom, 2020 | App | NR |
| Athilingam, 2016 & 2017 | App | NR |
| Goldstein, 2014, | App | NR |
| Vuorinen, 2014 | App + TM | NR |
| Seto, 2012 & 2012 | App + TM | NR |
| Heiney | App | “customized feedback (ie, clinical decision support) that sent an alert message if the weight exceeded standards set by the home health protocol for monitoring heart failure. The message instructed the participant to call the health care provider if they experienced an increase of 2-3 pounds in a day or over 5 pounds in a week.” |
| Guo, 2019 | App + TM | NR |
| Park, 2019 | App + TM | NR |
| Ware, 2019 | App + TM | Figure 1. Screens of the Medly app showing the incomplete morning card with required readings, the symptoms questionnaire, and **personalized** self-care feedback after all 4 readings were taken and processed by the algorithm |
| Foster, 2018 & 2018 | App | NR |
| Suthipong, 2018 | App | “The HFAA tracks the patient’s warning signs and symptoms to allow for early detection of worsening heart failure and assists the patient in calculating and administering diuretics. The app also prompts patients to begin a **customized** action plan when signs of deterioration are identified, which are differentiated using the red–yellow–green color system”.  “HF patients can utilize the information and resources provided by the HFAA that can help HF patients **personalize** their treatment by reporting changes in their health status every day (i.e., offering advice) and adjusting diuretic dosages”. |
| Alnosayan, 2017 | App + TM | NR |
| Radhakrishnan, 2016 | App | NR |
| **Qualitative studies** | | |
| Schmaderer, 2020 b | App | NR |
| Woods, 2019 | App | “A researcher spent 10-30 minutes providing an overview of the app interface, assisted with completing the **personalized** settings (dry weight, daily fluid restriction volume, daily step count aim, physical activity goals, and reminders), and determined self-management priorities based on patient preferences” |
| Portz, 2018 | App | NR |
| Sebern, 2018 | App | NR |
| Haynes, 2017 | App | NR |
| Srinivas, 2017 | App | “Accordingly, we engineered the back-end logic and server communication protocols so that prior behavior (e.g., trends in logged data) or outside intervention (e.g., by a clinician) could influence the cards selected or dealt over time. Given the identified need for **customization**, Engage [app] was designed to be flexible with respect to the content of the cards and to accommodate both a starter set of default cards as well as the opportunity to create custom card decks (e.g., per patient, per diagnosis, per clinic, etc.)” |

Abbreviations: App; mobile application; TM: telemonitoring.
