# Supplementary material for: The Use of Mobile Apps for Heart Failure Self-management: Systematic Review of Experimental and Qualitative Studies
Source: JMIR Cardio. 2022 Mar 31;6(1):e33839. doi: 10.2196/33839 (PMC9015755; doi:10.2196/33839)
Supplement: Multimedia Appendix 7 [file cardio_v6i1e33839_app7.docx]

## Multimedia appendix 7: Intervention features of included articles

| Author, year | Intervention | Devices connected (automatic transmission of data)^a^ | Vital signs (BP or HR) tracking | Symptom monitoring | Liquid tracking | Weight tracking | Data display (graphs, tables, charts) | Active feedback | Medication reminders / adherence | Physical activity support | Educational content |
| --- | --- | --- | --- | --- | --- | --- | --- | --- | --- | --- | --- |
| Clays, 2021 | App | Weight scale, BP/HR monitor | + (daily, with reminders) | - | - | + (daily, with reminders) | + | + | + | + | + |
| Schmaderer, 2021 a | App | Weight scale | - | - | - | + (daily, with reminders) | + | - | + | - | + |
| Wei, 2021 | App | Weight scale | - | + (triggered by weight gain) | - | + (daily, with reminders) | + | + | - | + | + |
| Yanicelli, 2021 | App + TM | - | + (daily, with reminders) | + (daily, with reminders) | - | + (daily, with reminders) | - | + | - | - | + |
| Rahimi, 2020 | App + TM | Weight scale, BP/HR monitor | + (daily, with reminders) | + (daily, with reminders) | - | + (daily, with reminders) | + | + | - | - | + |
| Wonggom, 2020 | App | - | - | - | - | - | - | - | - | - | + |
| Athilingam P, 2016 & 2017 | App | HR, accelerometer, physical activity | + (daily, with reminders) | + (daily, with reminders) | - | + (daily, with reminders) | + | + | + | + | + |
| Goldstein CM, 2014 | App | - | - | - | - | - | - | - | + | - | - |
| Vuorinen AL, 2014 | App + TM | - | + (weekly) | + (weekly) | - | + (weekly) | + | + | - | + | + |
| Seto E, 2012 & 2012 | App + TM | Weight scale, BP/HR monitor, ECG for those without ICD | + (daily, with reminders) | + (daily, with reminders) | - | + (daily, with reminders) | + | + | - | - | - |
| Heiney, 2020 | App | - | - | + | - | + (daily, with reminders) | + | + | - | - | + |
| Guo X, 2019 | App + TM | Weight scale, BP/HR monitor | + (daily) | + (daily) | - | + (daily) | + | - | + | - | - |
| Park C, 2019 | App + TM | Weight scale, BP/HR monitor | + (daily) | + (daily) | - | + (daily) | + | - | - | - | + |
| Ware P, 2019 | App + TM | Weight scale, BP/HR monitor | + (daily, with reminders) | + (daily, with reminders) | - | + (daily, with reminders) | + | + | - | - | - |
| Foster M, 2018 & 2018 | App | - | + (daily) | + (daily) | - | + (daily) | - | - | + | + | + |
| Suthipong C, 2018 | App | - | + (daily, with reminders) | + (daily, with reminders) | + | + (daily, with reminders) | - | + | - | - | + |
| Alnosayan N, 2017 | App + TM | Weight scale, BP/HR monitor, glucose | + (daily, with reminders) | + (daily, with reminders) | - | + (daily, with reminders) | + | - | + | + | + |
| Radhakrishnan K, 2016 | App | - | - | + (daily, with reminders) | - | - | - | - | + | + | + |
| Woods L, 2019 | App | - | + (daily) | + (daily) | + (daily) | + (daily, with reminders) | + | - | + | + | + |
| Portz JD, 2018 | App | - | - | + (daily) | - | + (daily) | + | - | - | - | - |
| Sebern MD, 2018 | App | - | - | - | - | - | - | - | - | - | + |
| Haynes SC, 2017 | App | - | + | + | - | + | - | - | + | - | - |
| Srinivas P, 2017 | App | - | + (daily) | + (daily) | + (daily) | + (daily) | + | - | + | + | + |

Frequency of monitoring is mentioned when reported by authors. Abbreviations: BP: blood pressure; ECG: electrocardiogram; HR: heart rate; ICD: implantable cardioverter defibrillator; +: present; -: absent. ^a^Wireless connection via Bluetooth in all studies except for one where it was not reported (Guo X, 2019).
