# Supplementary material for: The Use of Mobile Apps for Heart Failure Self-management: Systematic Review of Experimental and Qualitative Studies
Source: JMIR Cardio. 2022 Mar 31;6(1):e33839. doi: 10.2196/33839 (PMC9015755; doi:10.2196/33839)
Supplement: Multimedia Appendix 8 [file cardio_v6i1e33839_app8.docx]

## Multimedia appendix 8: Behavior Change Techniques present in the interventions of included articles

| **Author, year** | **Characteristics and BCTs of the intervention** | |
| --- | --- | --- |
| Clays, 2021 | **App + devices**  2.2 Feedback on behavior  2.3 Self-monitoring of behaviour  2.4 Self-monitoring of outcome of behaviour  2.7 Feedback on outcome of behaviour  3.3 Social support (emotional)  4.1 Instruction on how to perform the behaviour  7.1 Prompts/cues  8.1 Behavioral practice/rehearsal  --  2.2, 2.3 physical activity monitoring with wristband and feedback via app graph display; 2.4, 2.7 self-monitoring and display of weight, BP; 3.3 “providing psychological support (including elements of cognitive behavioural therapy and mindfulness exercises); 4.1 “lifestyle advice on nutrition"; “overall disease education about CHF and its treatment”; 7.1 “notification system for medication intake and self-monitoring; 8.1 “measure their BP, heart rate, and daily weight” | |
| Schmaderer, 2021 a | **App + wireless weight scale + zoom visit with clinicians**  2.3 Self-monitoring of behaviour  2.4 Self-monitoring of outcome of behaviour  2.7 Feedback on outcome of behaviour  4.1 Instruction on how to perform the behaviour  7.1 Prompts/cues  8.1 Behavioral practice/rehearsal  --  2.3 Record medication taking; 2.4 Record weight, 2.7 “Graphs review weekly weight trends.”; 4.1 “educational tips”; 7.1, 8.1 “reminders to take scheduled medication and weigh daily.” | |
| Wei, 2021 | **App + wireless weight scale**  2.3 Self-monitoring of behaviour  2.4 Self-monitoring of outcome of behaviour  2.7 Feedback on outcome of behaviour  4.1 Instruction on how to perform the behaviour  7.1 Prompts/cues  8.1 Behavioral practice/rehearsal  --  2.3 “Patients picked foods consumed at each meal from a list of available food items with corresponding sodium content, while patients entered length and mode of exercise performed each day.”; 2.4 “synced automatically with a Bluetooth-linked digital scale to record daily weight”; 2.7, “Depending on symptom severity, the app had an automated risk-based algorithm to instruct patients to contact study staff or contact their doctor”; 4.1, 7.1, 8.1 “The daily To-Do list prompted patients to watch the educational heart failure video of the day, exercise, weigh themselves, or complete other healthy activities in addition to 3 self-management functionalities: Track, Learn, and Coach; featured 13 interactive lessons including videos with topics recorded and narrated by cardiologists”.  Active feedback:  “Depending on symptom severity, the app had an automated risk-based algorithm to instruct patients to contact study staff or contact their doctor” | |
| Yanicelli, 2021 | **Telemonitoring via app**  2.4 Self-monitoring of outcome of behaviour  2.7 Feedback on outcome of behaviour  4.1 Instruction on how to perform the behaviour  7.1 Prompts/cues  8.1 Behavioral practice/rehearsal  --  2.4, 8.1 “that collects measurements of weight, blood pressure, heart rate and symptoms (checklist of questions about swelling in ankles, legs, shortness of breath etc.) daily”; 2.7 “and sends an alert to the participating physicians if a risky situation occurs (measurements are outside of normal ranges”; 4.1 “educational functionality that promotes self-learning about lifestyle, self-care and healthy habits of HF patients”; 7.1 reminders  Active feedback:  “Every day the same nurse reviewed patients’ data through the website and acted to all alert situations by contacting the patient for assessment and then, if necessary, contacted the participating physicians to receive advice about needed changes in medications or diet” | |
| Rahimi, 2020 | **Telemonitoring via tablet app + devices**  2.4 Self-monitoring of outcome of behaviour  2.7 Feedback on outcome of behaviour  4.1 Instruction on how to perform the behaviour  7.1 Prompts/cues  8.1 Behavioral practice/rehearsal  **--**  2.4; 8.1 “daily measurements of weight, blood pressure, and pulse.”; 2.7 “Home monitoring measures that are considered to be clearly abnormal as per current practice guidelines (ie, an increase in weight by 2–3 kg over2–3 d) are flagged, and participants receive immediate automated feedback via the tablet computer”; 4.1 “generic educational material such as animations and video clips on heart failure and strategies for managing it.”; 7.1, 8.1 “Depending on the participant's usage record, personalized messages are sent electronically to motivate them to engage in self-management activities”  Active feedback:  “Home monitoring measures that are considered to be clearly abnormal as per current practice guidelines (ie, an increase in weight by 2–3 kg over2–3 d) are flagged, and participants receive immediate automated feedback via the tablet computer to contact their physician or nurse for further advice. If no such flags are raised, participants receive a message at the end of their session to indicate that their readings are within an acceptable range.” | |
| Wonggom, 2020 | **App with avatar**  4.1 Instruction on how to perform the behaviour  --  4.1 “The content of the avatar app is based on the Heart Foundation of Australia's booklet, ‘Living Well with Heart Failure’ (National Heart Foundation of Australia, 2016) and includes four sections: (a) understanding heart failure; (b) looking after yourself; (c) things to do every day and; (d) emergency action plans.” | |
| Athilingam, 2017 | **App (HeartMapp) + Tracker (BioHarness-3 chest sensor)**  2.2 Feedback on behavior  2.3 Self-monitoring of behaviour  2.4 Self-monitoring of outcome of behaviour  2.6 Biofeedback  2.7 Feedback on outcome of behaviour  4.1 Instruction on how to perform the behaviour  5.1 Information about health consequences  7.1 Prompts/cues  8.1 Behavioural practice/rehearsal  - -  2.2, 8.1 “HeartMapp encourages walking 3 to 4 times a week and provides feedback to patients on performance”; 2.3 “BioHarness-3 was used to obtain heart rate and accelerometer data”; 2.4, 7.1 “tailored daily prompts and are provided access to the assessment window to check weight, blood pressure, and answer the short questionnaires on HF symptoms”; 2.6, 4.1 “HeartMapp is designed to teach patients about using biofeedback to attain 6 breaths per min and offer feedback on their performance”; 2.7 “On the basis of the information gathered, the HeartMapp provides an alert with the following feedback (…) the Yellow Zone (“take an extra dose of water pill if prescribed by your doctor”); 4.1, 5.1 “HeartMapp includes audio-enabled interactive teaching tools on the nature of heart failure, importance of low-salt diet, exercise regimen, HF medications, and managing other chronic diseases or conditions and feelings about HF as well as heart and brain connection”  Active feedback:  “The HeartMapp provides automated feedback; Green Zone (“no change – continue current self-management practices”); Yellow Zone (“take an extra dose of your water pill prescribed by your doctor and call doctor’s office for an early follow-up”); Orange Zone (“call the doctor’s office now”); and Red Zone (prompts to call 911).” | |
| Goldstein, 2014 | **App (iRx Reminder LLC)** (Intervention)  4.1 Instruction on how to perform the behavior  7.1 Prompts/cues  - -  4.1 “A medication adherence app provided medication reminders”; 7.1 “Participants could also view a list of their medications with information on each medication, including special instructions, such as taking it with a meal or before bedtime” | Eletronic pillbox (Medsignals)  (Comparator)  7.1 Prompts/cues  - -  7.1 “An electronic pillbox (Medsignals, Austin TX), the telehealth medication container, was used as one medication reminder device” |
| Vuorinen, 2014 | **App + weight scale + blood pressure meter (Telemonitoring)**  1.3 Goal setting (outcome)  2.4 Self-monitoring of outcome of behavior  2.7 Feedback on outcome of behavior  4.1 Instruction on how to perform the behavior  8.1 Behavioral practice/rehearsal  --  1.3, 2.7 “In the context of each submission of information, the patient received automatic machine-based feedback of whether the reported parameter was within their personal targets set by the nurse”; 2.4; 8.1 “The patients were advised to carry out and report the measurements [blood pressure, pulse, body weight] together with the assessment of symptoms once a week”; 4.1 “the patients were given a home-care package including a weight scale, a blood pressure meter, a mobile phone, and self-care instructions”  Active feedback:  “In the context of each submission of information, the patient received automatic machine-based feedback of whether the reported parameter was within their personal targets set by the nurse” – Example of the feedback sent in a screenshot “Great (BP) -go on same way) | |
| Seto, 2012 | **App + weight scale + blood pressure meter + ECG recording (Telemonitoring)**  1.3 Goal setting (outcome)  2.4 Self-monitoring of outcome of behavior  2.7 Feedback on outcome of behavior  4.1 Instruction on how to perform the behavior  7.1 Prompts/cues  8.1 Behavioral practice/rehearsal  --  1.3 “Clinicians were able to modify any necessary physiological target ranges per patient through a secure website”; 2.4 “Patients also answered symptom questions (mainly yes/no) through the mobile phones”; 2.7 “The mobile phone displayed an instruction on what to do after taking each measurement. A final message or alert based on the physiological and symptom information was sent to the mobile phone”; 7.1, 8.1 “If a patient did not take all the required measurements by 10 am each morning, an automated adherence reminder phone call was sent to their home telephone”; 4.1 “The patients in the telemonitoring group were given an individual training session on how to use the system […]weight and blood pressure readings[…] They were asked to use the telemonitoring system for 6 months to take daily morning weight and blood pressure readings”  Active feedback:  “The mobile phone displayed an instruction on what to do after taking each measurement. A final message or alert based on the physiological and symptom information was sent to the mobile phone” | |
| Heiney, 2020 | **App**  2.4 Self-monitoring of outcome of behavior  2.7 Feedback on outcome of behaviour  3.3 Social support  4.1 Instruction on how to perform the behavior  7.1 Prompts/cues  8.1 Behavioral practice/rehearsal  --  2.4, 8.1, 7.1 “reminder to weight every morning”; 2.7 “graphical display of data”; 3.3 “motivational messages”; 4.1 “educational message”; “The educational messages about heart failure self-management were derived from the heart failure teaching material provided to patients diagnosed with heart failure, and included information on diet, sleep, stress, and medication adherence.”  Active feedback  “customized feedback (ie, clinical decision support) that sent an alert message if the weight exceeded standards set by the home health protocol for monitoring heart failure. The message instructed the participant to call the health care provider if they experienced an increase of 2-3 pounds in a day or over 5 pounds in a week.” | |
| Guo, 2019 | **App + weight scale + blood pressure meter (Telemonitoring)**  2.4 Self-monitoring of outcome of behavior  2.7 Feedback on outcome of behavior  4.1 Instruction on how to perform the behavior  7.1 Prompts/cues  8.1 Behavioral practice/rehearsal  --  2.4 “Participants can use the app at home to record and upload comprehensive data elements related to the risk of CHF self-care management, including daily recording of symptom and sign changes and medication adherence”  2.7 “When data are outside an acceptable range, participants will receive video calls or text messages via the app”; 4.1 “participants can communicate with physicians more conveniently through their handheld mobile phone with the assistance of the remote consultations function of our app. They can send text messages to their general practitioners round the clock for help and receive personalized guidance on long-term self-care management of CHF via the mobile app”; 7.1 “reminders for tracking medical appointments and next visits are visible on the mobile app to keep track of their schedule”; 8.1 “they were reminded to use the app or browse the Web platform more than once a week.” | |
| Park, 2019 | **Apps (HealthPROMISE digital therapeutic and iHealth) + weight scale + blood pressure meter** (Telemonitoring)  2.4 Self-monitoring of outcome of behaviour  2.7 Feedback on outcome of behavior  4.1 Instruction on how to perform the behavior  7.1 Prompts/cues  8.1 Behavioral practice/rehearsal  - -  2.4, 4.1, 7.1 “Patients were provided with HF education content, SMS or text reminders, and the ability to track their patient-reported outcomes (PROs) via preselected symptom checkboxes”; 2.7 “Any critical red-flag values, for example, a greater than 2-pound weight gain within 24 hours or greater than 5-pound weight gain within a week automatically alerted the physician and prompted the patient to seek medical attention”; 8.1 “Patients were instructed to measure their weight and BP each day” | |
| Ware, 2019 | **App (Medly app) + weight scale + blood pressure meter (Telemonitoring)**  2.4 Self-monitoring of outcome of behavior  2.7 Feedback on outcome of behavior  they receive training on how to use the technology. A part of this training includes highlighting the importance of taking daily readings  4.1 Instruction on how to perform the behavior  7.1 Prompts/cues  8.1 Behavioral practice/rehearsal  --  2.4 “smartphone app that patients use to take weight, blood pressure, and heart rate readings as well as record their symptoms using a “yes/no” questionnaire”; 2.7 “if the algorithm identifies that key readings are out of range or that there is a worrying trend in weight gain, the app generates and displays self-care feedback messages which are highlighted in a different color depending on the determined urgency”; 4.1 “they receive training on how to use the technology. A part of this training includes highlighting the importance of taking daily readings”; 7.1 “an automated phone call to their primary phone line (personal mobile phone or home landline) to remind patients if they have not yet taken morning readings by 10 am”; 8.1 “Patients are instructed to take these 4 readings daily within 30 min of each other before 12 pm”  Active feedback:  “If the algorithm identifies that key readings are within the acceptable range, the reading values are presented on the screen with a message telling patients that their readings are fine on that day. However, if the algorithm identifies that key readings are out of range or that there is a worrying trend in weight gain, the app generates and displays self-care feedback messages which are highlighted in a different color depending on the determined urgency” | |
| Foster, 2018 | **App (HF App)**  2.4 Self-monitoring of outcome of behavior  4.1 Instruction on how to perform the behavior  --  2.4 “The daily statistics button led users to a Daily Quiz form that prompted entry of daily physiologic data including weight, blood pressure, pulse, and oxygen saturation”; 4.1 “ Tactical skills include strategies such as how to adhere to prescribed diets or medications, situational skills involve strategies such as how to decide whether to take an extra diuretic dose” | |
| Suthipong, 2018 | **App (HFAA app)**  2.4 Self-monitoring of outcome of behavior  2.7 Feedback on outcome of behavior  3.1 Social support (unspecified)  4.1 Instruction on how to perform the behavior  7.1 Prompts/cues  8.1 Behavioral practice/rehearsal  --  2.4, 8.1 “All participants measured and entered their blood pressure and weight, and observed their symptoms on the HFAA every day”; 2.7 “HFAA provided adjustment of an individual’s diuretic and fluid intake (…) in real time and offered recommendations for self-management relative to their daily body weight, signs, and symptoms”; 3.1 “The social networking feature of the HFAA offers benefits of social support for self-management”; 4.1 “This resource provides patients with important information, such as the definition, cause, and factors of HF, and the ability to conduct self-weighing, self-administered pitting edema tests, and self-tested blood pressure measurements”; 7.1, 8.1 “Patients are reminded to check their weight and answer a set of questions about their symptoms each morning”  Active feedback:  “HFAA provided adjustment of an individual’s diuretic and fluid intake, monitoring of warning signs” (…) “delivers individually tailored advice to HF patients”. “The app can also recommend several ways to fit the advice it offers into the patient’s regimen”  “users entered daily information on the app. (…) They then received daily diuretic doses, fluid intake, and individual daily plans or recommendations in one of three colors as previously described. They also saw recommendations on the reporting screen as the same diuretic and fluid reporting time | |
| Alnosayan, 2017 | **App + weight scale + blood pressure meter + glucose meter (Telemonitoring)**  2.4 Self-monitoring of outcome of behavior  2.7 Feedback on outcome of behavior  4.1 Instruction on how to perform the behavior  7.1 Prompts/cues  8.1 Behavioral practice/rehearsal  --  2.4 “The app consisted of 6 tabs for biometrics, symptoms, reminders, messages, blood glucose, and trend charts. The symptoms section of the mobile app used a set of questions”; 2.7 “A patient was at high-risk if any of the measurements were in the high-risk range. Alternatively, a patient was at medium-risk if none of the measurements were in the high-risk range but at least one measurement was in the medium-risk range. This component also sent reminders and messages to patients and alerts to nurses”; 4.1 “a pool of motivational and educational messages was elicited from the preventive care physician. Examples of these messages included: “limit your total sodium intake today to no more than 1500mg,” “replace your salt shaker with fresh lemons”; 7.1, 8.1 “Patients were asked to measure their weight, blood pressure, and blood glucose (if they had diabetes) […] Reminders were for missing data, whereas motivational and educational messages were allocated randomly and sent to patients daily” | |
| Radhakrishnan, 2016 | **App (Heart health game)**  4.1 Instruction on how to perform the behavior  7.1 Prompts/cues  8.1 Behavioral practice/rehearsal  10.3 Non-specific reward  --  4.1 “Incorrect responses [to quiz questions on the HF self-management content] were followed by the presentation of relevant content on the speciﬁc HF self-management skill and repetition of the content-related questions”; 7.1, 8.1 “appropriate HF self-management related behaviors were encouraged with daily reminder tips and questions on adherence of the HF self-management skills of sodium restriction (diet), medication adherence, physical activity, and symptom checks”; 10.3 “As an incentive designed to motivate players to repeatedly engage with the content, the casino slot game provided opportunities for HF patients to earn additional betting chips if they made correct responses to the content related questions” | |
| Schmaderer, 2020 b | **App + wireless weight scale + zoom visit with clinicians**  2.3 Self-monitoring of behaviour  2.4 Self-monitoring of outcome of behaviour  2.7 Feedback on outcome of behaviour  4.1 Instruction on how to perform the behaviour  7.1 Prompts/cues  8.1 Behavioral practice/rehearsal  --  2.3 Record medication taking; 2.4 Record weight, 2.7 “Graphs review weekly weight trends.”; 4.1 “educational tips”; 7.1, 8.1 “reminders to take scheduled medication and weigh daily.” | |
|  |  | |
| Woods L, 2019 | **App (Care4myHeart) + step counter**  1.1 Goal setting (behavior)  1.3 Goal setting (outcome)  2.2 Feedback on behavior  2.3 Self-monitoring of behaviour  2.4 Self-monitoring of outcome of behaviour  2.7 Feedback on outcome of behavior  4.1 Instruction on how to perform the behavior  7.1 Prompts/cues  8.1 Behavioral practice/rehearsal  --  1.1, 1.3 “The *Home screen* provides a shortcut to the priority *My Plan* icons based on patient goals and a reminder summary. The *My Plan* section includes nine important components of self-management of heart failure: medications, symptoms, exercise, weight, fluid, well-being, diet, blood pressure and pulse, and future plans”; 2.2, 2.3 The 7-day step counter graph provided an accurate picture of the mobility status to patients who used the feature”; 2.4, 2.7 “For daily weight management, graphs were deemed useful, accurate, and relevant and provided feedback to users, as viewing 7-day weight trends heightened self-awareness.”; 4.1 “Participants did not watch the instructional exercises videos due to disinterest”; 7.1 “Reminders, alerts, infographics, videos, health professional advice, and information pages throughout the app aim to guide patients to manage their heart failure”; 8.1 “there was potential benefit to heart failure self-management for *daily management of illness* with the benefits of accurately recording and reviewing personal health data” | |
| Portz JD, 2018 | **App (HF app)**  2.4 Self-monitoring of outcome of behaviour  2.7 Feedback on outcome of behavior  8.1 Behavioral practice/rehearsal  --  2.4, 2.7, 8.1 “The HF app was designed to allow older adults with HF to record their weight, log their symptoms, and symptom severity (…) How much do you weight today? – Graph displaying the weight trend” | |
| Sebern MD, 2018 | **App (iSCIP)**   - 1. Action plan   3.2 Social support (practical)  3.3 Socal support (emotional)  4.1 Instruction on how to perform the behavior  5.1 Information about health consequences  --  1.4 “A prototype app was developed to assess and display the care values, preferences and subsequent care planning. (…) They use the app’s care plan feature to discuss what resources they have now, and what may be helpful for them in the future. The care plan identifies tasks that could be delegated, when more assistance is needed”; 3.2, 3.3 “we added additional care preferences tasks specific to HF self-management, such as preparing meals that follow dietary restrictions, symptom monitoring, physical activity, walking, climbing stairs, assistance with falls, assistance with appearance and being available 24 hrs”; 4.1, 5.1 “the iSCIP incorporates mobile computing devices (iPads), a custom iPad application (commonly referred to as an “app”), and links to network educational resources designed for partners and clinicians. The handheld iPad platform offers direct visual and tactile interaction without the need to manage a separate display, keyboard and mouse; this allows users to focus on concepts and tasks, rather than the mechanics of interacting with the device” | |
| Haynes SC, 2017 | **App (OnPoint application)**  2.4 Self-monitoring of outcome of behaviour  7.1 Prompts/cues  8.1 Behavioral practice/rehearsal  --  2.4 “Other features included care team contacts, tracking weight/blood pressure”; 7.1, 8.1 “The prototype focused on the medication reconciliation and medication taking, priorities identified by both patients and providers: constructing a comprehensive medication list, creating a medication schedule, filling a pillbox, and tracking medications. (…) For instance, because patients often use pillboxes to manage medications, we created an interface that evokes a pillbox metaphor to connect the digital representation on the tablet to the patient’s physical pillbox. The novel medication management features allow a user to (…) sorts medications into appropriate day/time compartment in the rendered physical pillbox, which all serve to help the patient organize medications accurately and efficiently.” | |
| Srinivas P, 2017 | **App (Engage)**  1.1 Goal setting (behavior)  1.3 Goal setting (outcome)  2.2 Feedback on behavior  2.3 Self-monitoring of behaviour  2.4 Self-monitoring of outcome of behaviour  2.7 Feedback on outcome of behavior  4.1 Instruction on how to perform the behavior  5.1 Information about health consequences  10.3 Non-specific reward  --  1.1, 1.3 “GOAL” or action planning daily behaviors based on longer term goals (…) Participants performed seven types of tasks: (…) setting goals, checking if a goal was met, reviewing long-term goals”; 1.1, 2.3 “Today’s plan: 30 min walk. Report what you actually did”; 2.4, 4.1 “LOG” or logging information on symptom data (e.g., weight) and self-care behavior (e.g., taking medication); “HINT” or reading of practical hints related to heart failure and performing heart failure self-care (…)The hint function was meant to address self-care knowledge deficits, particularly by suggesting practical ways to relate and apply knowledge”; 2.2, 2.7 “two feedback loops: one corresponding to the patient’s direct use of Engage” (Figure 6: progress report on weighting, graphs on diet and colour code for achievement of the plan); 5.1 “provide an opportunity for patients to gain self-care knowledge by engaging in self-care behavior in parallel with the delivery of informational content”; 10.3 “A motivational incentive was added in the form of virtual coins that are earned with each card played and could optionally be redeemed for tangible rewards.” | |
